# Recent Progress on Zinc-Ion Rechargeable Batteries

**DOI:** 10.1007/s40820-019-0322-9

**Published:** 2019-10-17

**Authors:** Wangwang Xu, Ying Wang

**Affiliations:** Department of Mechanical and Industrial Engineering, Louisiana State University, Baton Rouge, LA 70803 USA

**Keywords:** Zinc-ion batteries, Electrolyte, Cathode, Zinc anode, Flexible device

## Abstract

The increasing demands for environmentally friendly grid-scale electric energy storage devices with high energy density and low cost have stimulated the rapid development of various energy storage systems, due to the environmental pollution and energy crisis caused by traditional energy storage technologies. As one of the new and most promising alternative energy storage technologies, zinc-ion rechargeable batteries have recently received much attention owing to their high abundance of zinc in natural resources, intrinsic safety, and cost effectiveness, when compared with the popular, but unsafe and expensive lithium-ion batteries. In particular, the use of mild aqueous electrolytes in zinc-ion batteries (ZIBs) demonstrates high potential for portable electronic applications and large-scale energy storage systems. Moreover, the development of superior electrolyte operating at either high temperature or subzero condition is crucial for practical applications of ZIBs in harsh environments, such as aerospace, airplanes, or submarines. However, there are still many existing challenges that need to be resolved. This paper presents a timely review on recent progresses and challenges in various cathode materials and electrolytes (aqueous, organic, and solid-state electrolytes) in ZIBs. Design and synthesis of zinc-based anode materials and separators are also briefly discussed.

## Highlights

- The recent progress about zinc-ion batteries was systematically summarized in detail, including the merits and limits of aqueous and nonaqueous electrolytes, various cathode materials, zinc anode, and solid-state zinc-ion batteries.
- Current challenges and perspectives to future research directions are also provided.

## Graphical abstract

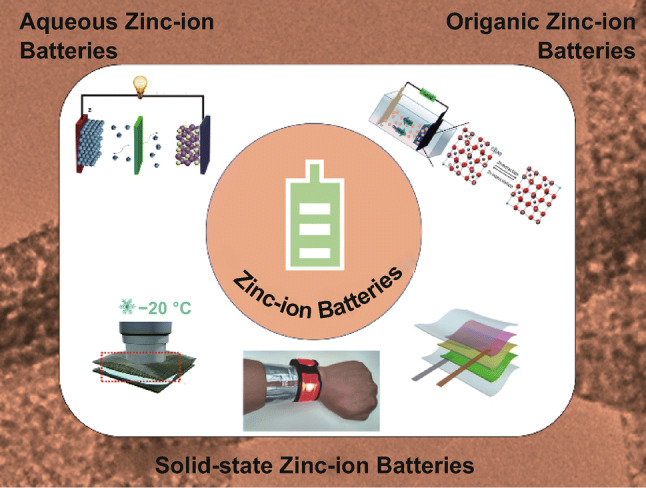

## Introduction

Energy crisis and environmental pollution have become two of the most serious issues in the present time [1–4]. For hundreds of years, fossil fuels (petroleum, coal, etc.) have dominated the energy supply for the needs of humanity [5–8]. However, usage of fossil fuels can lead to numerous environmental issues, especially air pollution, which is caused by the emissions of sulfur dioxide, nitrous oxide, carbon dioxide, and other gases containing volatile organic compounds [9, 10]. Meanwhile, the development of environment-friendly energy sources such as solar cell and wind electricity is seriously restrained by their intermittent production and inability for storing energy [11, 12]. To overcome the above-mentioned challenges, electrical energy storage (EES) offers an effective way to improve the reliability and scalability of the grid [13–15]. In recent years, much progress has been made by developing new energy technologies, especially rechargeable batteries [16–19]. Since the first secondary cell (lead–acid battery) was invented about 150 years ago, numerous kinds of rechargeable batteries have been designed, such as nickel zinc battery, nickel metal hydride, and lithium-ion batteries (LIBs) [20, 21]. For several decades, lithium-ion batteries have been widely applied as commercial energy storage devices owing to their advantages of high efficiency in delivering energy, high voltage, and long cycling life [22, 23]. However, many issues such as high cost and safety problems seriously hinder the large-scale applications of lithium-ion batteries [24, 25]. In recent years, a lot of research work focused on aqueous rechargeable batteries using naturally abundant monovalent ions (Na^+^, K^+^) and multivalent cations (Zn^2+^, Mg^2+^, Al^3+^) as charge carriers [26–29]. The aqueous zinc-ion batteries (ZIBs) are very appealing owing to the unique properties of zinc anode, including the low cost, rich resources of zinc metals, high chemical/physical stability, environmental friendliness, and high safety [28, 30–35].

A typical ZIB consists of a cathode for hosting Zn ions, zinc metal anode, electrolyte, and a separator to separate cathode and anode, which is quite similar to the structure of a LIB. Zinc ions are moving between cathode and anode during charging and discharging processes. Since Volta et al. used zinc metal as electrode in battery in 1999 for the first time, Zn metal has been deemed as an ideal anode material in types of primary and secondary Zn cells due to many excellent properties, especially high capacity of Zn metal anode, nontoxicity, relatively low redox potential (− 0.76 V vs. standard hydrogen electrode (SHE)), high safety, and low cost. Therefore, such Zn metal has been applied in various batteries, such as Ni–Zn batteries, MnO_2_–Zn batteries, Zn-ion batteries, and Zn–air batteries. Among them, Zn–MnO_2_ batteries are dominant as primary batteries owing to the properties of low cost and high energy density [36, 37]. Later effort further expands the alkaline Zn–MnO_2_ batteries to secondary battery field, while, as electrode in rechargeable batteries, the continuous formation of zinc dendrite in zinc metal and the irreversible reaction lead to limited cycling life and low discharge capacity. Several decades ago, Yamamoto et al. first designed aqueous zinc-ion batteries by replacing the alkaline electrolyte with aqueous mild acid zinc sulfate electrolyte [38]. Unlike the case in alkaline Zn–MnO_2_ batteries, the formation of by-products (ZnO, Zn (OH)_2,_ etc.) on zinc metal is very few. Also, compared with Mg metal, another advantage of zinc metal is that zinc can be dissolved more easily in electrolyte and deposited more easily on metal anode. In recent years, a lot of research work has been focused on both zinc metal anode and cathode materials, especially manganese-based oxides, vanadium-based oxides, polyanion compounds, sustainable quinone analogs, and Prussian blue analogs. This research work has made significant progress on the development of ZIBs [39–44].

Furthermore, the rapid development of various wearable electronic devices keeps promoting the exploration on portable energy storage devices with superior electrochemical performance and desirable mechanical flexibility [45, 46]. As a promising candidate, rechargeable solid-state zinc-ion storage systems also have increasingly attracted research interests [30, 47]. It should be noted that safety problem requires serious consideration in the wearable and flexible devices while they maintain high energy storage capabilities, because these electronics may undergo continuous mechanical force or damage such as strike or being bended, and directly in contact with human body [48]. In comparison with liquid electrolytes, solid-state electrolytes show a noticeable advantage for avoiding the electrolyte leakage. Furthermore, solid-state electrolytes show high stability, desirable flexibility, and can effectively control the formation of zinc dendrite and the dissolution of active electrode materials in zinc-ion storage systems [49]. To date, electrolytes based on zinc sulfate (ZnSO_4_) and zinc triflate (Zn(CF_3_SO_3_)_2_) in conjunction with polymer hosts such as ZnSO_4_–gelatin, Zn(CF_3_SO_3_)_2_–polyethylene oxide, and Zn(CF_3_SO_3_)_2_–polyvinyl alcohol have been reported [50–53]. However, these polymer electrolytes suffer from insufficient mechanical strength, undesirable ionic conductivity, and fast degradation. By employing these electrolytes, the batteries display relatively narrow voltage window and undesirable cycling stability, posing challenges for the gel electrolyte selection and preparation method. Additionally, an intrinsically self-healable ZIB is proposed by the utilization of hydrogel electrolyte, in order to effectively enhance the recoverability of the devices against various shape deformations [51]. More importantly, the exploration of desirable solid-state electrolytes with a wide working range from subzero to high temperature is still lacking and researchers need to put more efforts to promote its development, which can boost the practical applications of flexible ZIBs used in airplanes, aerospace, or ocean vehicles. Consequently, the careful design on the suitable solid-state electrolytes with high stability, excellent zinc-ion conductivity, superior mechanical properties, and easy fabrication is highly required for the further development of flexible and safe rechargeable zinc-ion based energy storage devices, which could effectively prevent electrolyte leakage issue and achieve industrial production of batteries with desirable structures [30].

Recently, Fan et al. have analyzed and summarized the Zn-ion storage mechanisms of various cathode materials [54]. And Liu et al. summarized the design strategies for vanadium-based cathodes for aqueous ZIBs [13]. Though many review articles have been published in recent years [53], few review papers discuss various electrolytes in the ZIBs in details despite that electrolyte is a crucial component in ZIBs and there have been rapid new developments concerning it lately. The main difference in this review is that we systematically present recent progresses and challenges of all the components in ZIBs in detail, including various cathode materials, aqueous and nonaqueous electrolytes, solid-state ZIBs, and Zn anodes. We also provide opinions on current limitations of ZIBs and perspectives for future research directions. The review is divided into six parts: (1) research background, recent development and research progress of ZIBs based on (2) aqueous electrolytes, (3) summarization of representative cathode materials, (4) solid-state zinc-ion batteries, (5) design of zinc anodes and separators, and (6) current challenges and perspectives for future research directions.

## Zinc-Ion Batteries based on Aqueous Electrolytes

Aqueous rechargeable batteries are regarded as promising candidate for large-scale energy storage due to their high safety nature, low cost, and environmental friendliness [55–57]. Moreover, compared with organic electrolyte, the aqueous electrolytes can provide two times higher ionic conductivities (~ 1 S cm^−1^) due to the higher mobility of ions in water environment [56]. In addition, the rechargeable zinc-ion batteries employing divalent ions can provide higher specific capacity as well as energy density than monovalent ions due to the double electrons involved in redox reactions. Because the most common anode material in ZIBs is zinc metal and the battery capacity is more limited by cathode than anode, a vast variety of cathode materials have been explored for applications in high-performance ZIBs, as detailed below.

### Manganese Oxide Cathode Materials

For thousands of years, manganese-based materials have been widely used by humans due to their abundant resources in the crust. Manganese oxides have remarkable diversity of atomic structures and multivalent phases due to different oxidation states of Mn: + 2, + 3, and + 4. Therefore, manganese oxides can be widely applied as catalysts, battery materials, and deoxidizer in steel making, etc. Moreover, manganese oxides are the first material researched and reported as cathode in the field of ZIBs due to its high valence state and specific phases which can readily accommodate inserted ions. To better understand manganese oxides and their crystal structures and the Mn-based materials applied in ZIBs, we summarize the recent studies about manganese oxides below.

#### *γ*-MnO_2_

**Fig. 1:**
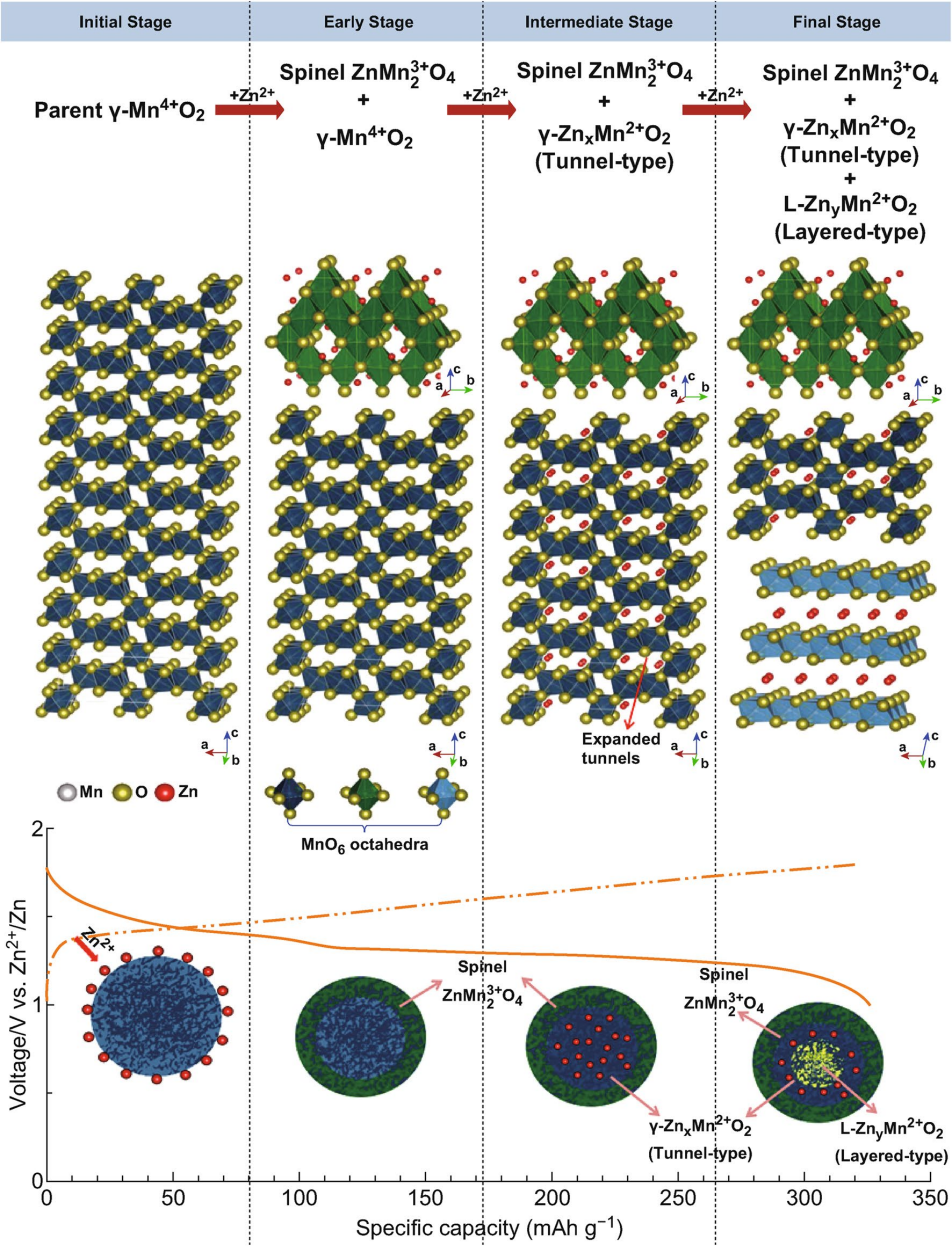
Schematic illustration of the transformation process of *γ*-MnO_2_ cathode with Zn^2+^ ion insertion [58]. With permission from American Chemical Society

Kim et al. investigated the phase evolution in a mesoporous *γ*-MnO_2_ material during the intercalation of zinc ions via in situ Synchrotron XANES and XRD [58]. The results confirm that with the intercalation of zinc ions, the typical tunnel-type *γ*-MnO_2_ gradually transferred to spinel-type ZnMn_2_O_4_ and two new intermediary Mn(II) phases, namely tunnel-type *γ*-Zn_*x*_MnO_2_ and layered-type L-Zn_*y*_MnO_2_. With the extraction of zinc ions, most of these phases return back to the *γ*-MnO_2_ phase, indicating reversible phase transformation during electrochemical reaction (Fig. 1). At the current density of 0.05 mA cm^−2^, the mesoporous *γ*-MnO_2_ cathode can deliver an initial discharge capacity of 285 mAh cm^−2^.

#### α-MnO_2_

The *α*-MnO_2_ is also a hot topic to be applied as cathode in ZIBs due to its large and stable 2 × 2 tunnels structure, which can accommodate the intercalated Zn^2+^ ions. The *α*-MnO_2_ was first studied as cathode of ZIBs by Zeng et al. in 2009 with the initial capacity of 210 mAh g^−1^ [59]. The capacity can be almost 100% maintained after 100 cycles at current density of 6 °C, showing reversible zinc storage and robust 2 × 2 tunnel structure during electrochemical reactions. However, the electrochemical reaction mechanism for MnO_2_/Zn batteries is still a discussion topic until now. Currently, three mechanisms are proposed in the aqueous *α*-MnO_2_/Zn batteries: zinc intercalation/deintercalation, conversion reaction, and H^+^ and Zn^2+^ co-insertion.

*Zinc intercalation/deintercalation mechanism* Due to the small radius of zinc ions, various compounds with layered or tunnel structure enable the intercalation of zinc ions. In 2012, Kang et al. investigated electrochemical mechanism of *α*-MnO_2_/Zn batteries with aqueous electrolyte [32]. They confirmed that ZnMn_2_O_4_ was formed after the insertion of Zn^2+^ ion into the tunnels of *α*-MnO_2_ and reversibly returned to *α*-MnO_2_ after the extraction via ex situ XRD, and they proposed the zinc intercalation/deintercalation mechanism as below [32]:

1 $${\text{Cathode:\;}}{\text{Zn}}^{2 + } + 2{\text{e}}^{-} + 2\,\alpha {\text{{-}MnO}}_{2} \leftrightarrow {\text{ZnMn}}_{ 2} {\text{O}}_{4}$$

2 $${\text{Anode:}}\;{\text{Zn}} \leftrightarrow {\text{Zn}}^{2 + } + 2{\text{e}}^{ - }$$

Afterward, some studies focus on the phase transformation after the intercalation of zinc ions in tunnels of *α*-MnO_2_. For example, Oh et al. revealed that the electrochemical reaction process of *α*-MnO_2_/Zn involves a reversible phase transition between tunneled (*α*-MnO_2_) and layered Zn–buserite, instead of spinel structure ZnMn_2_O_4_ [33].

*Conversion reaction mechanism* In 2016, Liu et al. significantly improved the electrochemical performance of Zn/MnO_2_ battery by adding MnSO_4_ additive in mild ZnSO_4_ aqueous electrolyte [60]. They found that the MnSO_4_ additive can suppress the Mn^2+^ dissolution into the electrolyte, leading to improved rate performance and capacity retention capability. In addition, to understand the electrochemical behavior, they further investigated the morphology and phase evolution of *α*-MnO_2_ material via TEM and STEM-EDS mapping. The results reveal that the electrochemical mechanism of MnO_2_ is based on the conversion reaction between MnOOH and MnO_2_ instead of Zn^2+^ ion intercalation/deintercalation in/out of MnO_2_, which is formulated as below [60]:

Cathode:

3 $${\text{H}}_{ 2} {\text{O}} \leftrightarrow {\text{H}}^{ + } + {\text{OH}}^{ - }$$

4 $$\alpha {\text{{-}MnO}}_{2} + {\text{H}}^{ + } + {\text{e}}^{ - } \leftrightarrow {\text{MnOOH}}$$

5 $$1/2{\text{Zn}}^{2 + } + {\text{OH}}^{ - } + 1/6{\text{ZnSO}}_{4} + \, x/6{\text{H}}_{ 2} {\text{O}} \leftrightarrow 1/6{\text{ZnSO}}_{4} \left[ {{\text{Zn}}\left( {\text{OH}} \right)_{2} } \right]_{3} \cdot x{\text{H}}_{ 2} {\text{O}}$$

6 $${\text{Anode:}}\;1/2{\text{Zn}} \leftrightarrow 1/2{\text{Zn}}^{2 + } + {\text{e}}^{ - }$$

The MnOOH phase is confirmed by XRD at the fully discharged state. Instead of intercalation of zinc ions, the reaction is induced by the reaction between *α*-MnO_2_ and inserted protons. Moreover, another electrochemical mechanism of H^+^ and Zn^2+^ co-insertion was reported.

*The H*^+^ *and Zn*^*2*+^ *co*-*insertion mechanism* It is possible that the open tunnel *α*-MnO_2_ enables the co-insertion of H^+^ and Zn^2+^ ions. To understand the mechanism, Wang et al. [61] developed Zn/MnO_2_ batteries and studied consequent H^+^ and Zn^2+^ insertion processes by using electroanalytical technology combined with XRD, SEM, and TEM. The two insertion processes happened at two different voltage regions. The galvanostatic intermittent titration technique (GITT) results show that the H^+^ insertion process happens at region I with low overvoltage of 0.08 V, and the Zn^2+^ insertion process takes place at region II with much higher overvoltage of 0.6 V. The significant difference of H^+^ and Zn^2+^ insertion processes is mainly attributed to the different resistances for H^+^ and Zn^2+^ ion diffusion. The EIS results show that the ohmic resistances of these two regions are close, while the charge transfer resistance of region II is much larger than that in region I. Moreover, the bivalent zinc ions have much larger radius and stronger electrostatic interactions with the host atoms than H^+^ ions, leading to slower Zn^2+^ diffusion. It can be also observed in ZIBs with other cathodes, such as V_2_O_5_·*n*H_2_O [65] and NaV_3_O_8_·1.5H_2_O [78]. Despite that three different Zn^2+^ storage mechanisms in MnO_2_/Zn batteries have been proposed, more efforts are still needed to further identify the electrochemical reaction mechanism and further enhance the battery performance.

Many strategies have been applied to improve the performance of MnO_2_/Zn batteries. For example, to slow down the manganese dissolution during electrochemical cycling, Liang et al. introduced potassium ions and oxygen defects in MnO_2_ [62]. The incorporated K^+^ ions can stabilize the Mn-based cathodes, and the oxygen defects can improve electrical conductivity and diffusion rate for zinc ions. Therefore, the K_0.8_Mn_8_O_16_/Zn batteries show improved capacity at high rate. At a high current density of 1 A g^−1^, the K_0.8_Mn_8_O_16_/Zn batteries can provide a high specific capacity over 300 mAh g^−1^ and a high energy density of 398 Wh kg^−1^ for over 1000 cycles, showing outstanding durability and energy density. Composite nanostructure design is another effective strategy to boost the performance of MnO_2_/Zn batteries. For example, Xia et al. designed the manganese dioxide with polyaniline preintercalated in the interlayer space. The intercalated polyaniline can strengthen the layered structure of manganese dioxide in nanoscale size [63]. Furthermore, the combination of PANI-reinforced layered structure and nanoscale particle size (~ 10 nm) can efficiently hinder the phase transformation induced by the insertion of hydrated H^+^/Zn^2+^ ions, maintaining the structural stability during the electrochemical reaction. The co-insertion process of hydrated H^+^ and Zn^2+^ ions was also carefully examined and clarified a self-regulating mechanism involving generation/dissolution of electrolyte (zinc hydroxide sulfate). The PANI-reinforced MnO_2_ achieves a high capacity of 125 mAh g^−1^ for over 5000 cycles, showing long cycling life with high capacity.

#### ZnMn_2_O_4_

Inspired by the success of LiMn_2_O_4_, spinel MFe_2_O_4_ (M = Zn, Ni, or Cu), Co_3_O_4_, ZnCo_2_O_4_, and ZnMn_2_O_4_ have also been explored as electrode material for batteries. Manthiram et al.’s study investigated the reaction mechanism of the insertion process of Zn^2+^ ions in spinel compositions ZnMn_2−*x*_Ni_*x*_O_4_ (*x* = 0, 0.5, and 1) [64]. The zinc ions can be extracted from the structure in acid condition during the Mn^3+^ disproportionation reaction, while, with the increase in Ni content, the extraction of Zn^2+^ will decrease. The researching results appear that the spinel-structured materials are not quite suitable for the intercalation of zinc ions, while, with a certain content of defects (vacancies), the Zn-ion diffusion can be much easier due the lower electrostatic repulsion. Inspired by the understanding that the creation of defects in spinel materials can open additional pathways for the transportation of divalent ions. Chen and his co-workers prepared the cation-defective ZnMn_2_O_4_ spinel as the host material for intercalation of Zn^2+^ cations [19]. They applied ZnMn_2_O_4_/carbon composite as the cathode material and studied the electrochemical reaction mechanism via XRD, Raman, FTIR, NMR, and electrochemical measurements. They demonstrated that the abundant cation vacancies and small nanoscale size can facilitate the charge transfer and Zn^2+^ insertion into ZnMn_2_O_4_ spinel structures. The ~ 100% Zn plating/stripping efficiency enables long cycling life with high capacity. At high current of 500 mA/g, the ZnMn_2_O_4_ spinel carbon composite material can supply the specific capacity of 150 mAh g^−1^ for 500 cycles with retention of 94%.

### Vanadium-Based Cathode Materials

#### V_2_O_5_

Building by sharing edges and corners of square pyramids chains, the vanadium pentoxide shows square pyramid-layered structure. More importantly, square pyramid layer of *α*-V_2_O_5_ can include water molecules or ions such as Na and Zn ions into the interlayers, which may change the layered structure and significantly affect the discharge/charge processes and electrochemical performances of ZIBs.

**Fig. 2:**
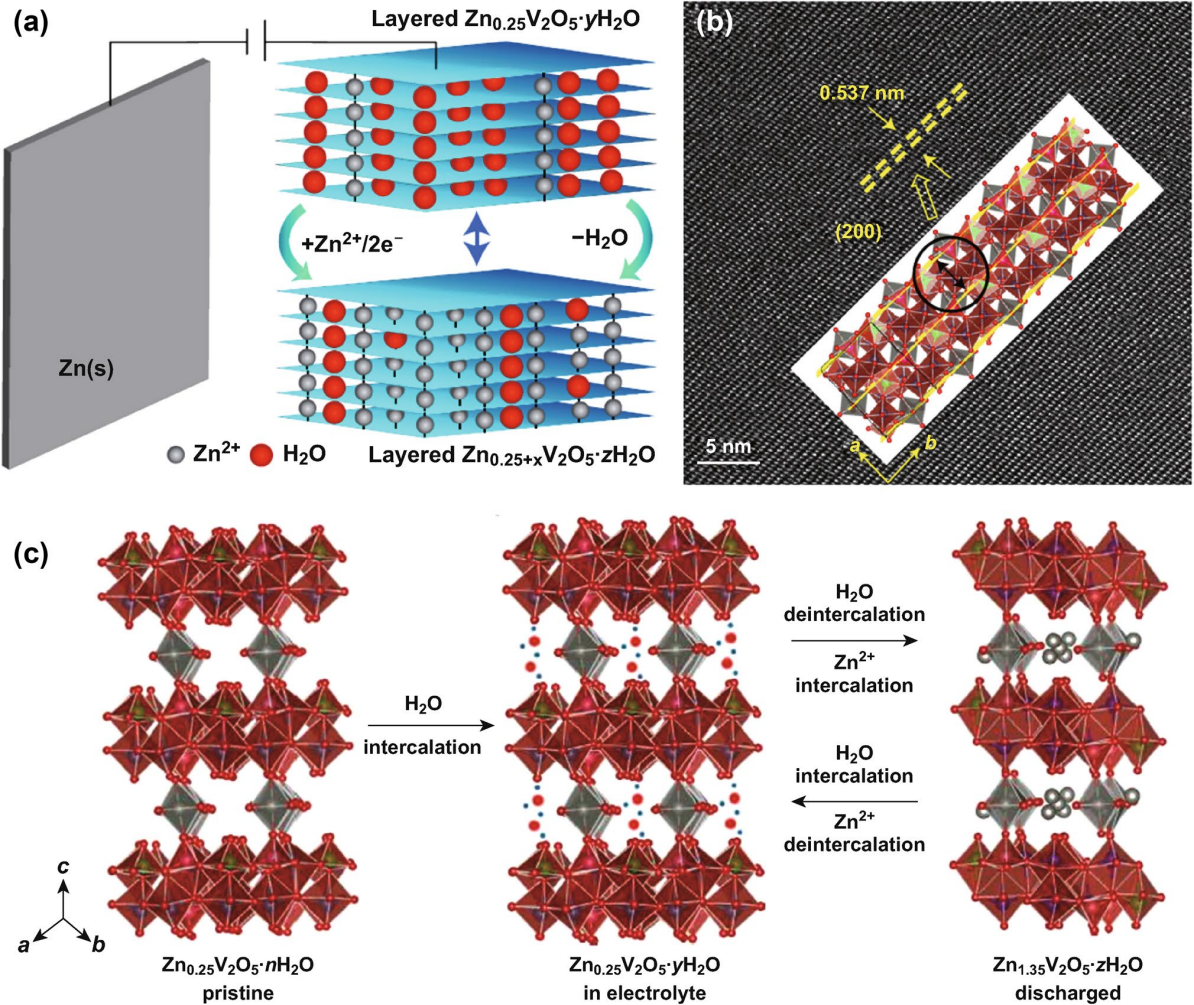
a Schematic of aqueous ZIBs based on Zn_0.25_V_2_O_5_ cathode. Due to the water molecules between the V_2_O_5_ layers, the interlayer space expands. **b** The related HRTEM image of the Zn_0.25_V_2_O_5_·*n*H_2_O nanobelts. **c** Schematics showing the co-intercalation of water molecules accompanying Zn^2+^ in/out of the interlayer space of V_2_O_5_ layers during charging and discharging process [67]. With permission from Springer Nature

In 2018, Cheng et al. investigated the Zn storage mechanism in commercial V_2_O_5_ cathode material using Zn(CF_3_SO_3_)_2_ aqueous electrolyte [65]. It is reported that Zn^2+^ cations can reversibly insert/extract through the layered structure of commercial V_2_O_5_ bulks. Moreover, it is interesting to find that the bulk V_2_O_5_ morphology gradually develops into porous nanosheet structure after cycling, which is caused by the exfoliation during charging and discharging processes (Fig. 2). As a result, the V_2_O_5_ porous nanosheets can deliver a very high reversible capacity of 372 mAh g^−1^ at current density of 5 A g^−1^ for over 4000 cycles. In addition, the co-intercalated H_2_O molecules can enhance the transportation of Zn^2+^ ions. Subsequently, Mai et al. systematically studied the critical role of structural H_2_O on Zn^2+^ intercalation into the layered structure of V_2_O_5_·*n*H_2_O [66]. They found that water molecules can dramatically work as a “lubricant” to promote the fast transportation of zinc ions. Specifically, the structural water can function like a charge screening media in redox reactions during cycling, which can not only increase the interlayer distance, but also decrease electrostatic interactions of H_2_O-solvated Zn^2+^ ions in the V_2_O_5_ framework. This result was confirmed by the solid-state magic-angle spinning results. Benefited from the “lubricating” effect of structural water, V_2_O_5_·*n*H_2_O can deliver a very high energy density of ~ 144 Wh kg^−1^ at 0.3 A g^−1^, which is comparable with LiCoO_2_/graphite batteries.

The critical role of structural water molecules and interlayer-doped ions is also verified by many researchers. For example, Linda et al. first studied Zn_0.25_V_2_O_5_·*n*H_2_O as the positive electrode for Zn cells in 2016 (Fig. 2a) [67]. They found that water molecules intercalated in the interlayers can buffer the high charge density of zinc ions and reduce the activation energy for charge transfer at the interface of cathode material (Fig. 2b). The indigenous Zn ions in Zn_0.25_V_2_O_5_·*n*H_2_O crystals can stabilize the layered structure, leading to the long cycling stability. Moreover, the stable layered structure can release the stress generated by the insertion/extraction of zinc ions during charging/discharging process and also short the pathways for the transportation of zinc ions (Fig. 2c). At the rate of 1 °C, the Zn_0.25_V_2_O_5_·*n*H_2_O can provide the capacity up to 300 mAh g^−1^, with energy density of ~ 450 Wh l^−1^ and capacity retention of more than 80% for over 1000 cycles.

#### V_*x*_O_*y*_ Cathode Materials

**Fig. 3:**
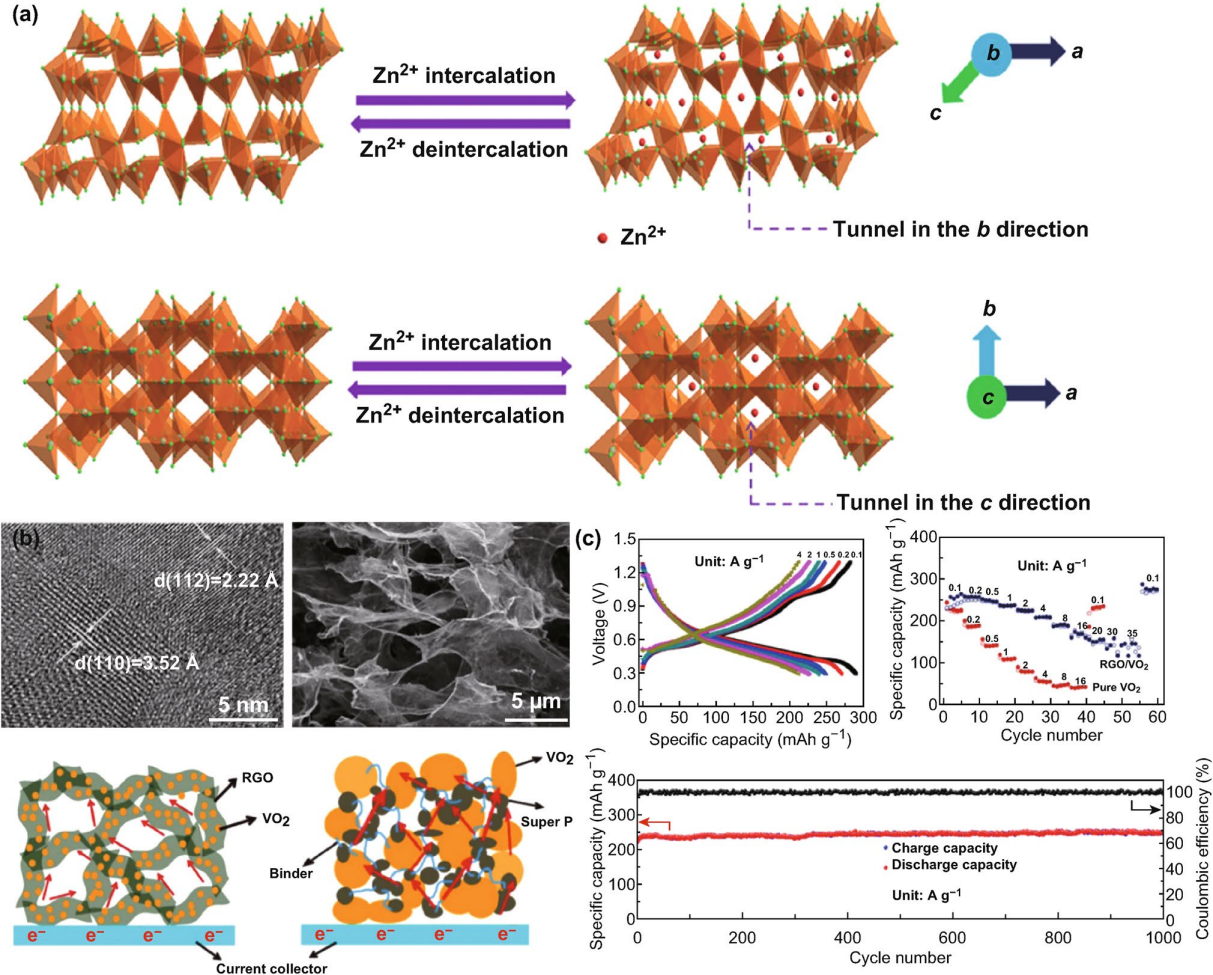
a Schematic illustration of Zn^2+^ intercalation/deintercalation in VO_2_ crystals. **b** HRTEM and SEM images of RGO-VO_2_; schematic view of the pathways for electron transportation in different samples (RGO-VO_2_ and VO_2_-super P). **c** Electrochemical performance of RGO-VO_2_ composite film, including charge/discharge profiles, rate capability, and long-term cycling test at 4 A g^−1^ [67]. With permission from Elsevier and Wiley

Constructed by distorted VO_6_ octahedra by sharing corners and edges, vanadium dioxide has a special tunnel-like framework. The big tunnel-like framework can facilitate the transportation for the diffusion of inserted ions. In recent years, VO_2_ (B) has been widely studied as potential electrode material in organic electrolytes with monovalent ions, while the investigation of VO_2_ (B) in divalent/multivalent ion batteries is almost blank. In 2018, Yang et al. first introduced VO_2_ (B) as cathode material in aqueous ZIBs [68]. They investigated the electrochemical mechanism by in situ XRD combined electrochemical analyses. And the results demonstrate that VO_2_ (B) nanofibers have an intercalation pseudocapacitance behavior. The pseudocapacitance behavior is owing to the unique tunnel-like framework, which can not only provide efficient pathways for transporting Zn^2+^ ions, but also has strong mechanical stability during the intercalation/deintercalation of Zn^2+^ ions. Therefore, the VO_2_ (B) nanofibers show remarkable electrochemical performance. At the high current density of 300 °C, VO_2_ (B) nanofibers show the reversible capacity as high as 171 mAh g^−1^, high energy density of 297 Wh/kg, and power densities of 180 W kg^−1^. In addition, Niu et al. designed the freestanding reduced graphene oxide/vanadium dioxide (RGO/VO_2_) composite films to further improve the performance of VO_2_ as cathode for zinc-ion batteries. Zn/RGO/VO_2_ batteries exhibit an energy density of 65 Wh kg^−1^ even at a high power density of 7.8 kW kg^−1^ (Fig. 3). The superior performance attributes to the porous structure of RGO/VO_2_ composite film, which can provide efficient transportation of electrons and ions during cycling. Moreover, porous network can also release the stress and accommodate the volume change during the intercalation/deintercalation of Zn^2+^ into/from the VO_2_ crystals. In recent years, Wang et al. found an interesting phase transformation phenomenon during electrochemical process of VO_2_ [69]. They demonstrated that the monoclinic VO_2_ gradually transfers to bilayered V_2_O_5_·*n*H_2_O, which is induced by the initial insertion/extraction of zinc ions in VO_2_ during cycling. The phase transformation leads to significantly enlarged interlayer spacing and enhanced structural stability, enabling an improved battery performance: at the current density of 100 mA g^−1^ (corresponding to a specific energy density of 271.8 Wh kg^−1^), it can deliver a capacity of 274 mAh g^−1^ with an excellent capacity retention of 79% for over 10,000 cycles, showing a promising application as cathode material of ZIBs.

**Fig. 4:**
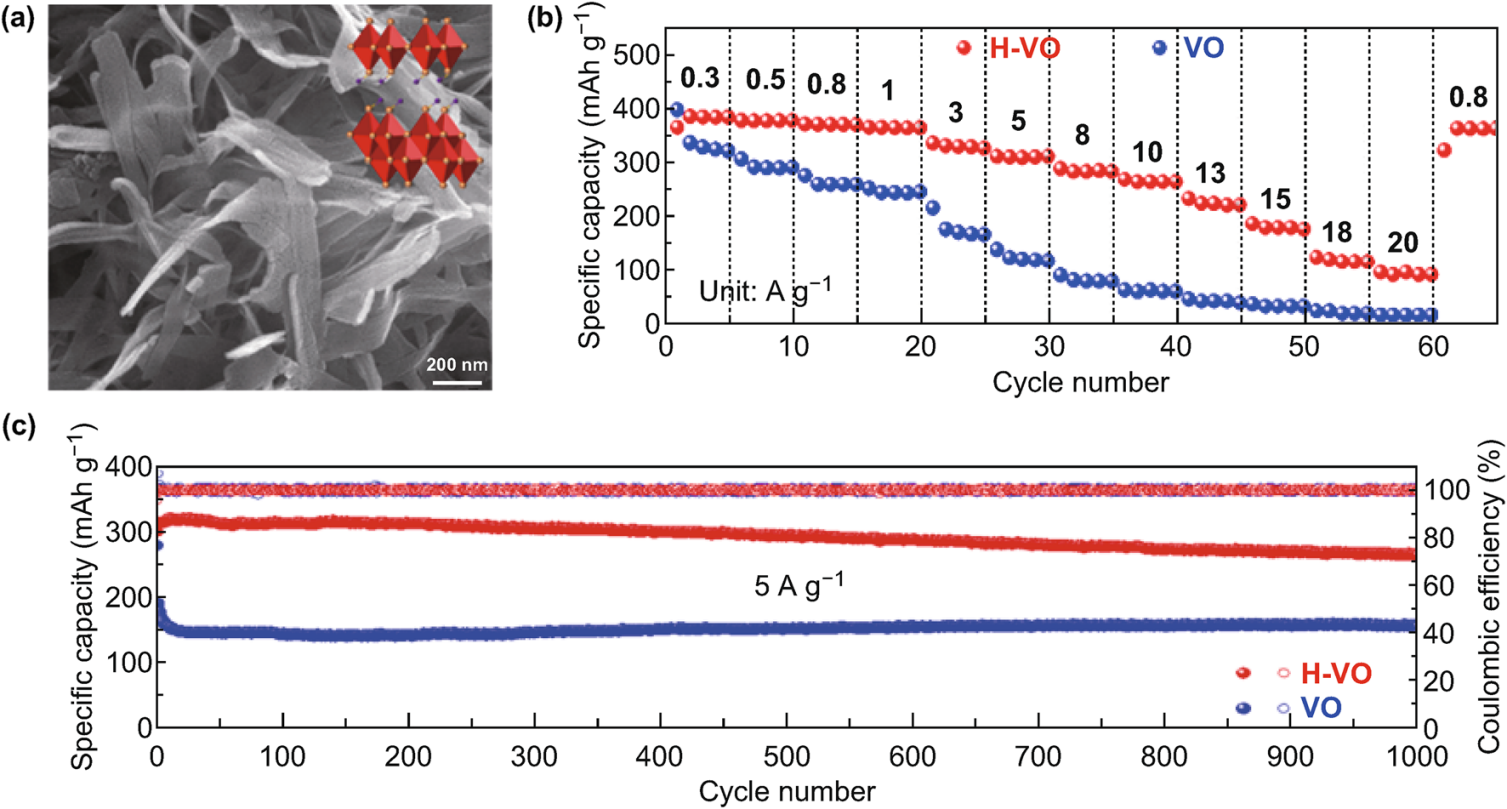
**a** SEM image of V_6_O_13_·*n*H_2_O (insert: the corresponding atomic structure). **b** Rate capabilities of V_6_O_13_·*n*H_2_O and V_6_O_13_ electrodes. **c** Long-term cycling performances of V_6_O_13_·*n*H_2_O and V_6_O_13_ cathodes at 5 A g^−1^ [70]. With permission from American Chemical Society

Interlayer-expanded V_6_O_13_·*n*H_2_O (H–VO) nanosheets were also prepared and evaluated as cathode material for aqueous ZIBs [70]. Benefiting from the enlarged interplanar spacing with abundant accessible channels and the ultra-thin nanosheet architecture with short diffusion pathway for Zn^2+^ migration, the H–VO cathode presents superior electrochemical property (Fig. 4). The as-synthesized H–VO nanosheets exhibit a very high and reversible capacity of 395 mAh g^−1^ at a current density of 0.1 A g^−1^, an exceptionally high rate capability up to 20 A g^−1^ with a decent capacity of 97 mAh g^−1^, and over 87% capacity retention after 1000 cycles, while the annealed V_6_O_13_ (VO) without lattice water shows much inferior electrochemical behaviors. This demonstrates the essential role of crystalline water in enhancing the electrochemical performances of vanadium compounds as cathode in aqueous ZIBs.

Choi et al. further elucidated the importance of water co-insertion with Zn^2+^ by the combination of density functional theory (DFT) calculations and experiments, rendering the high performance of V_6_O_13_ material as aqueous ZIB cathode [71]. Such intercalation mechanism showed that the co-inserted water can facilitate Zn^2+^ diffusion through reducing effective charge and thus provide electrostatic shielding. Benefiting from the hydrated intercalation, V_6_O_13_ presented a reversible capacity of 360 mAh g^−1^ at 0.2 A g^−1^, high rate capability up to 24 A g^−1^ with a decent capacity of 145 mAh g^−1^, and satisfactory cycling stability with 92% capacity retention after 2000 cycles.

Orthorhombic-structured V_3_O_7_·H_2_O nanowires were also studied as the cathode for ZIBs. Wei et al. designed aqueous ZIBs that composed with V_3_O_7_·H_2_O/rGO composite as the cathode and Zn-coated rGO as the anode [72]. The reduced graphene boosts the conductivity of both cathode and anode materials, leading to excellent electrochemical performance. It can deliver a high power density of 8400 W kg^−1^ at energy density of 77 Wh kg^−1^.

#### M_*x*_V_*y*_O_*z*_ Vanadate Cathode Materials

Enlarging the interlayer spacing via preintercalation of cations is an effective way to improve the electrochemical performance of electrodes for aqueous ZIBs. In recent years, vanadium-based nanowires intercalated with different cations (M_*x*_V_*y*_O_*z*_, M = H, Li, Na, K, Zn, Ca, Ag, Mn, etc.) have been widely studied. For example, Mai et al. designed the aqueous ZIBs based on H_2_V_3_O_8_ nanowire cathode. H_2_V_3_O_8_ nanowires can deliver a high capacity of 423.8 mAh g^−1^ at 0.1 A g^−1^, with capacity retention as high as 94.3% for over 1000 cycles [73]. The remarkable performance is owing to the layered structure of H_2_V_3_O_8_ with large interlayer spacing, which facilitates the transportation of zinc ions due to lower resistance and enables the intercalation/deintercalation of zinc ions with a slight change on structure. Moreover, Wang et al. further enhance the performance via designing H_2_V_3_O_8_ nanowires/GO composite [74]. The composite exhibits superior zinc-ion storage capability. At the current density of 1/3 °C, the composite shows a high capacity of 394 mAh g^−1^. At the high rate of 20 °C, the composite can deliver the capacity of 270 mAh g^−1^ (high power density of 2215 W kg^−1^) with a retention of 87% for over 2000 cycles, exhibiting remarkable performance at high rate. The excellent performance is owing to the synergistic merits of H_2_V_3_O_8_ nanowires and highly conductive graphene framework.

Lithium-ion intercalation is also an effective way to improve the electrochemical performance of V_2_O_5_. Liang et al. synthesized Li_*x*_V_2_O_5_·*n*H_2_O by the chemical intercalation of Li^+^ into the interlayer of V_2_O_5_·*n*H_2_O through the combination of hydrothermal and annealing processes [75]. The Li^+^ ions in V_2_O_5_·*n*H_2_O enlarge its interlayer spacing, thus facilitating the Zn^2+^ ion diffusion. Therefore, compared with the poor performance of the V_2_O_5_·*n*H_2_O electrode without preintercalation of lithium ions, the Li^+^ intercalated V_2_O_5_·nH_2_O electrode can deliver a high initial capacity of 304 mAh g^−1^ at current density of 5 A g^−1^, with stable retained capacity of 232 mAh g^−1^ after 500 cycles. Another material, Li_1+*x*_V_3_O_8_ (LVO), is also an interesting candidate to facilitate the intercalation/deintercalation of multivalent ions with large radius owing to the layered-type structure and vanadium in high oxidation states. Kim and his co-workers first applied the layered-type LiV_3_O_8_ as cathode for ZIBs and studied the detailed phase evolution during the intercalation of Zn ions via simulation techniques, in situ XRD and electrochemistry analysis [76]. The results reveal that after the intercalation of zinc ions, ZnLiV_3_O_8_ phase gradually transferred to reversible solid–solution Zn_*y*_LiV_3_O_8_ (*y* > 1) phase. The unique phenomenon leads to improved electrochemical performance. At the current density of 133 mA g^−1^, it can deliver a high specific capacity of 172 mAh g^−1^ for over 65 cycles with 75% capacity retention and 100% Coulombic efficiency. Another similar material, K_2_V_6_O_16_·2.7H_2_O (KVO), was also investigated by Kim and his group members. They studied the K_2_V_6_O_16_·2.7H_2_O nanorods as cathode materials in ZIBs for the first time [77]. The K_2_V_6_O_16_·2.7H_2_O compound has a unique structural arrangement of V_3_O_8_ layers of VO_6_ and V_2_O_8_ units with interstitial hydrated K ions. Moreover, ex situ XRD and XPS results showed that the diffusion behavior of Zn^2+^ ions in and out of the layered KVO structure is dominated by the redox reaction of vanadium. At the specific power of 72 W kg^−1^, the KVO nanorods can deliver a reversible high specific energy of 172.1 Wh kg^−1^ with ~ 82% capacity retention for over 500 cycles, indicating high energy density and long-term cyclability.

Na_0.33_V_2_O_5_ is composed by quadruple octahedra chains, which are linked by double chains of square pyramids via sharing their corners [78]. Due to the limited space for intercalated Zn^2+^ ions, Na_0.33_V_2_O_5_ framework suffers a huge structural stress after the intercalation of Zn^2+^ ions. After the intercalation of zinc ions, a new Zn_*x*_Na_0.33_V_2_O_5_ phase appeared due to the crystal distortion with Zn^2+^ intercalation. Therefore, a large capacity fading for about 100 mAh g^−1^ was observed during the initial two cycles due to the “dead sites” for inserted zinc ions. Sodium ions in sodium vanadium oxides are believed to act as pillars to stabilize the framework to keep the reversible phase transform during cycling. In the most recent years, many researchers focused on the development of sodium vanadium oxides with stable ion storage properties. For example, Chen and his co-workers further studied the electrochemical mechanism of NaV_3_O_8_·1.5H_2_O by ex situ Fourier transform infrared spectroscopy and nuclear magnetic resonance [79]. Their results confirm that accompanying with insertion/extraction of zinc ion, simultaneous proton insertion/extraction also exists during the electrochemical processes, which is different from conventional rechargeable batteries. This unique co-intercalation behavior ensures the remarkable electrochemical performance. At the current density of 50 mA g^−1^, it can deliver a high reversible specific capacity of 380 mAh g^−1^ (corresponding to energy density of 300 Wh kg^−1^) with capacity retention of 82% for over 1000 cycles, showing excellent zinc storage capability.

**Fig. 5:**
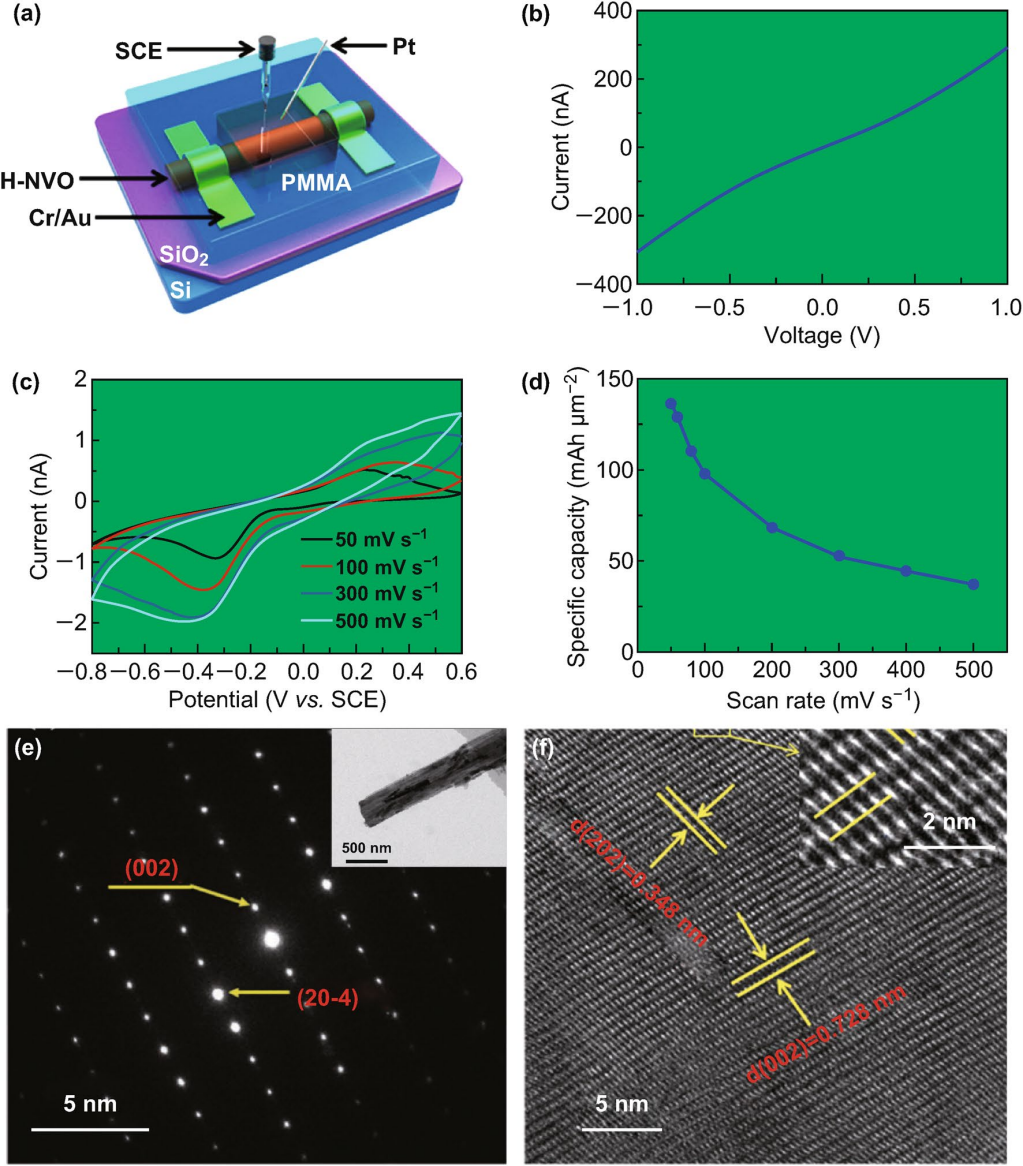
**a** Schematic view of single-nanowire ZIB, **b** the transport property, **c** the CV curves tested at different scan rates ranging from 50 to 500 mV s^−1^, **d** the specific capacitances at different scan rates [80], **e** the SAED pattern; the inset shows the TEM image and **f** HRTEM image of Na_0.33_V_2_O_5_ electrode after 100 cycles [78]. With permission from American Chemical Society and Wiley

The significant role of structured water in Na_2_V_6_O_16_ enhancing the electrochemical performances is further confirmed by Mai et al. [80] They designed a highly durable zinc-ion battery system with a Na_2_V_6_O_16_·1.63H_2_O nanowire cathode with an aqueous Zn(CF_3_SO_3_)_2_ electrolyte. Compared with only 17% of capacity retention of NaV_3_O_8_ nanowires at 5000 mA g^−1^ after 4000 cycles, the Na_2_V_6_O_16_·1.63H_2_O nanowires exhibit a capacity retention of 90% for over 6000 cycles at current density of 5000 mA g^−1^. Moreover, they assembled a single-nanowire-based zinc-ion battery to investigate the intrinsic Zn^2+^ storage mechanism at nanoscale (Fig. 5). The single-nanowire zinc-ion battery verifies the high electrical conductivity and current carrying capacity of Na_2_V_6_O_16_·1.63H_2_O. The layered structure of Na_1.1_V_3_O_7.9_@rGO is firstly employed as cathode for aqueous zinc-ion battery by Liang et al. [81]. The designed pilotaxitic Na_1.1_V_3_O_7.9_ nanoribbons/graphene composite shows improved performance. In addition, to confirm the structural stability of Na_0.33_V_2_O_5_ nanowires after long cycles, He et al. performed TEM characterization on the electrodes after cycling. Figure 5e, f shows the SAED pattern, HRTEM image of Na_0.33_V_2_O_5_ electrode after 100 cycles. The results confirm that the nanowire morphology and single crystalline structure are well maintained after long cycles. Another cation, Mn(II), can also act as robust structural pillars to stabilize the layered structure of hydrated vanadate [82]. Cao et al. reported manganese expanded hydrated vanadate as a cathode for ZIBs. The chemical insertion of Mn(II) cations can expand the interplanar spacing to 12.9 Å, leading to the reduced electrostatic interactions. In addition, the expanded interplanar spacing can facilitate fast intercalation of Zn ions at higher current densities, leading to significantly enhanced reversibility and cycling stability. At a specific current of 4 A g^−1^, manganese expanded hydrated vanadate can deliver a high specific capacity of 260 mAh g^−1^ for over 2000 cycles with a high capacity retention of 92%.

Alshareef et al. first reported another new material, layered Ca_0.24_V_2_O_5_ bronze, as the cathode material for aqueous ZIBs [83]. The prepared Ca_0.24_V_2_O_5_ is very suitable as cathode material for stable aqueous Zn^2+^ ion storage. At 0.2 C, the Ca_0.24_V_2_O_5_ structure can deliver a high capacity of 340 mAh g^−1^. At power density of 53.4 W kg^−1^, the Zn/Ca_0.24_V_2_O_5_ cells show a remarkable energy density of 267 Wh kg^−1^ for over 5000 cycles, exhibiting very high energy density and long cycling stability. Also, the low cost of Ca, Zn, V enables Ca_0.24_V_2_O_5_ a viable cathode for aqueous ZIBs in large-scale applications. Another novel material, Ag_0.4_V_2_O_5_, was synthesized by Liang et al. and first applied as cathode for ZIBs. They studied the phase evolution after the discharging process and demonstrated the displacement/intercalation mechanism [84]. Specifically, after the initial discharging, most of Ag^+^ in Ag_0.4_V_2_O_5_ is replaced by Zn^2+^, forming Zn_2_(V_3_O_8_)_2_. The formed Zn_2_(V_3_O_8_)_2_ can accommodate more inserted Zn^2+^ ions. The generated highly conductive Ag^0^ matrix within the material can support high electronic conductivity. Therefore, the prepared Ag_0.4_V_2_O_5_ material exhibits good rate capability and long cycling stability. At the high rate of 20 A g^−1^, it can still provide the stable capacity of 144 mAh g^−1^ for over 4000 cycles.

A series of layered ammonium vanadates, including NH_4_V_4_O_10_, NH_4_V_3_O_8_, and (NH_4_)_2_V_3_O_8_ with corresponding interlayer distance of 0.98, 0.79, and 0.56 nm, respectively, were examined as cathode materials for aqueous ZIBs [85]. Owing to the largest interlayer spacing of 0.98 nm and intercalated NH_4_^+^ as strong “pillars,” NH_4_V_4_O_10_ showed high energy density of 374.3 Wh kg^−1^ with the power density of 9000 W kg^−1^, and negligible capacity loss over 1000 cycles at 10 A g^−1^. Notably, the cycling stability of NH_4_V_4_O_10_ cathode was tested under high (50 °C) and low temperature (0 °C) at 5 A g^−1^, exhibiting a high reversible capacity of 377 mAh/g at 50 °C and decent capacity of 179 mAh g^−1^ at 0 °C, indicating impressive electrochemical properties working in a wide temperature range.

### Prussian Blue Materials

Prussian blue is a kind of prototype material with open framework structure, which contains zeolitic water and possesses special physical and chemical properties. In the typical Prussian blue material (KFe^3+^Fe^2+^(CN)_6_), the Fe^3+^ ions and Fe^2+^ ions are octahedrally connected with the nitrogen ends and carbon ends of the CN^−^ groups, respectively. One half of the open sites in framework structure are occupied by K^+^ ions. With the intercalation of more K^+^ ions, some of the Fe^3+^ ions are reduced to Fe^2+^. The color changes from blue to colorless. The yielded product is called Everitt’s salt. With the K^+^ ions extracted from Prussian blue, Fe^2+^ ions are oxidized. The color turns to yellow, and the product is called Prussian yellow.

#### CuHCF and ZnHCF Cathode Materials

As a typical Prussian blue analog-based material (PBA), copper hexacyanoferrate (CuHCF) has a well-studied open framework. Mantia et al. first applied CuHCF as a positive electrode in aqueous ZIBs. They found that CuHCF can provide an extremely high average potential of 1.73 V. At a current density of 1 °C, CuHCF can supply 90% of the theoretical capacity for 100 cycles with retention of 96.3% [86]. The battery also shows a high rate capability, delivering 96.1, 90, and 78 mAh g^−1^ at current density of 2.5, 5, and 10 °C, respectively. Moreover, Wang et al. prepared CuHCF nanocubes for application as cathode electrode in aqueous ZIBs [87]. They found that the intercalation/deintercalation of Zn^2+^ ions were controlled by the solid-phase diffusion of Zn^2+^ in/out of the CuHCF electrode. Mantia et al. further studied the long-term cycling stability of CuHCF in kinds of zinc-ion electrolytes [88]. They discovered that the anions and the concentration of zinc ions played a significant role for the stability of the cathode electrode. Basically, the stability of CuHCF electrode is higher with the lower concentration of electrolyte. This is due to the phase transition of the CuHCF, instead of the dissolution of the active material.

Liu et al. carefully studied the effect of different insertion cations (Na^+^, K^+^, and Zn^2+^) on the electrochemical reaction of ZnHCF [89]. ZnHCF can exist stably in the condition of aqueous ZnSO_4_ electrolyte, while ZnHCF would be dissolved in aqueous Na_2_SO_4_/K_2_SO_4_ electrolytes. Meanwhile, they discovered that the intercalation potential was highly dependent on ionic radius. Specifically, larger ionic radii can lead to higher charging/discharging potential. The ZnHCF-based ZIBs show an extremely high average operation voltage of 1.7 V, leading to a high specific energy density of 100 Wh kg^−1^.

### Other Cathode Materials

**Fig. 6:**
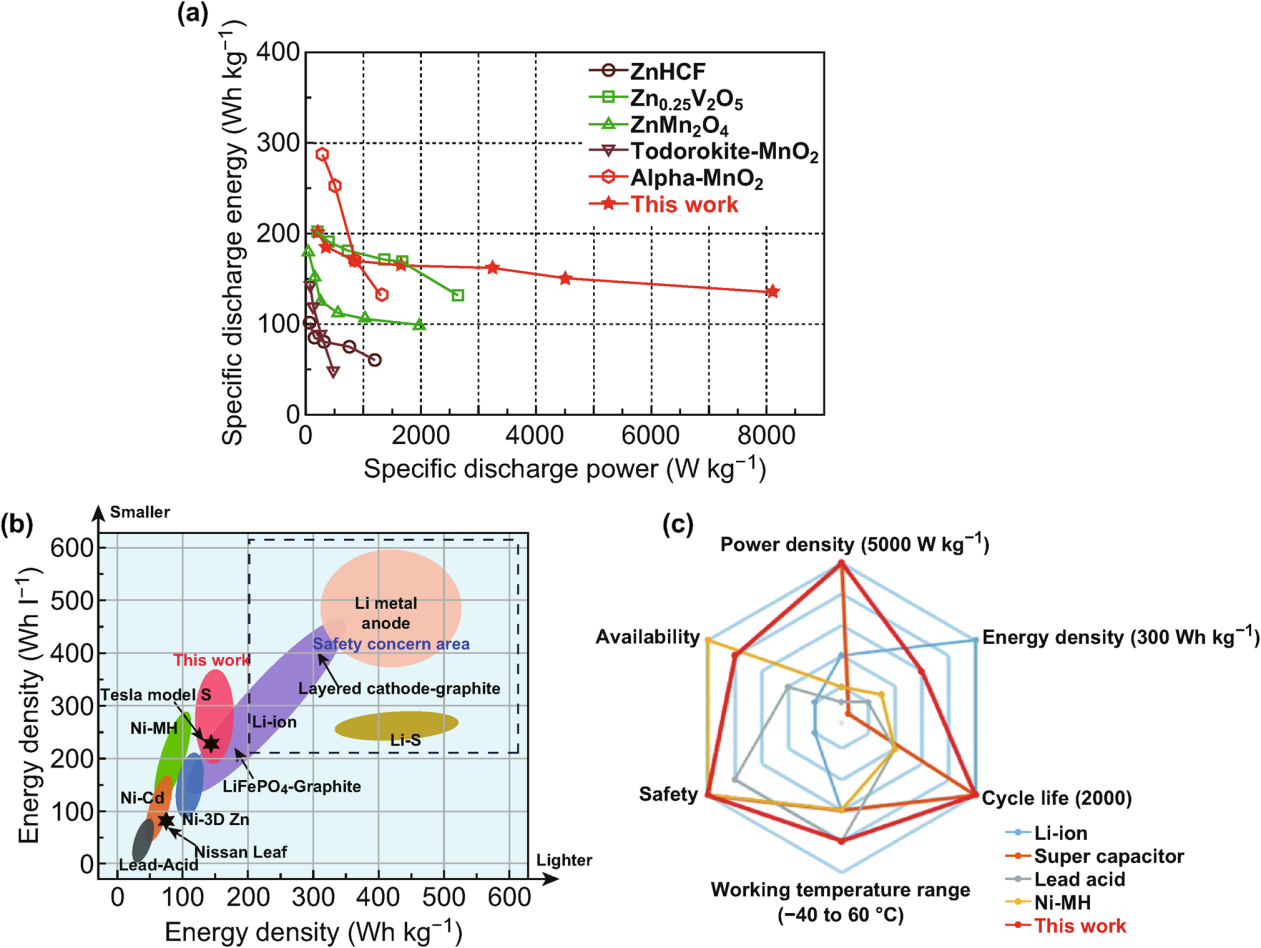
Comprehensive electrochemical performance for LiV_2_(PO_4_)_3_. **a** Comparison of LiV_2_(PO_4_)_3_ cathode with other researched cathode materials for ZIBs. **b** Gravimetric (Wh kg^−1^) and volumetric (Wh L^−1^) energy densities for different battery systems. **c** The spider chart for the itemized comparison of Zn/LiV_2_(PO_4_)_3_ cell with other commercial systems [90]. With permission from The Royal Society of Chemistry

The large family of polyanionic materials has been widely studied as cathodes for monovalent metal ion batteries (Li^+^ and Na^+^ ion batteries), due to their high redox voltage, plenty of vacancies to accommodate the inserted metal ions, as well as stable framework that is favorable for long cycling performance. In 2018, Wang et al. first designed a Zn/LiV_2_(PO_4_)_3_ battery and verified that the robust polyanion crystal structure could also enable the reversible intercalation of Zn^2+^ ions (Fig. 6) [91]. They demonstrated that the intercalated Zn^2+^ ions can be delocalized by multiple atoms through the *p–d* hybridization between the V-*d* and O-*p* orbitals. The Zn/LiV_2_(PO_4_)_3_ batteries can deliver the high voltage of 1.7 V and support both high power density and high energy density for over 4000 cycles. Huang et al. first introduced Na_3_V_2_(PO_4_)_3_ as cathode material for ZIBs in 2016 [43, 91]. They fabricated Na_3_V_2_(PO_4_)_3_ with carbon nanosheets wrapped around as intercalation host for zinc cations. The Zn/Na_3_V_2_(PO_4_)_3_ batteries exhibited excellent rate capability and long cycling life. At a current density of 0.5 °C, Na_3_V_2_(PO_4_)_3_ can deliver the capacity of 97 mAh g^−1^ for over 100 cycles. They claimed an ion occupying variation mechanism for the intercalation of zinc ions in polyanion crystal structure, which is also confirmed by CV and XRD results. Moreover, Chen et al. studied the electrochemical reaction mechanism of both Li_3_V_2_(PO_4_)_3_ and Na_3_V_2_(PO_4_)_3_ as cathodes materials in ZIBs [92]. They found that the crystal structure of both Li_3_V_2_(PO_4_)_3_ and Na_3_V_2_(PO_4_)_3_ is very stable in zinc-ion electrolyte. They further studied the effects of the pH value of aqueous electrolyte on electrochemical performance. They discovered that aqueous electrolyte with weak acidic pH value (4.0–4.5) can support the optimized electrochemical performance. Jiang et al. further investigated the electrochemical performance of Na_3_V_2_(PO_4_)_2_F_3_ as cathode electrode in ZIBs [93]. To avoid the formation of dendrite on Zn anode, they coated the zinc metal with a thin carbon film. The designed Zn/Na_3_V_2_(PO_4_)_2_F_3_ batteries can support a high average potential (1.62 V), high energy density (97.5 Wh kg^−1^) and a long cycling life for over 4000 cycles with capacity retention of 95%.

**Fig. 7:**
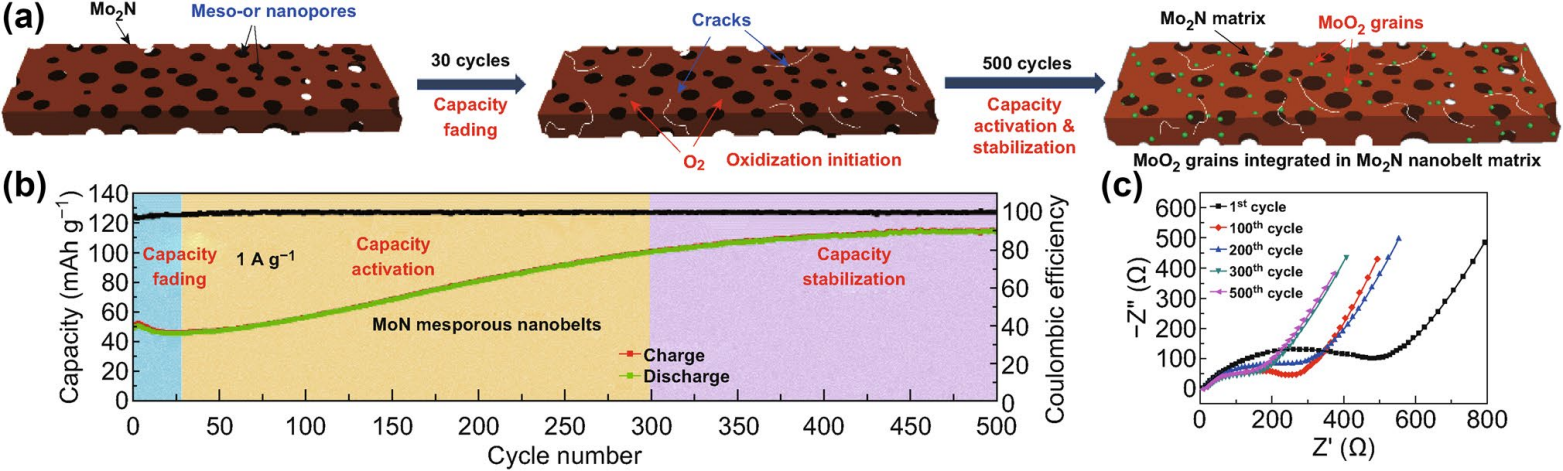
**a** Schematic of formation of MoO_2_/Mo_2_N composite materials during the electrochemical activation process. **b** The capacity during the electrochemical activation cycling process. **c** The Nyquist plots at different cycling times [95]. With permission from Elsevier

There have also been some studies on Mo-based electrode materials in ZIBs. For instance, MoO_3_ and MoS_2_ cathodes were found to deliver high initial discharge specific capacities up to 200 and 110 mAh g^−1^, respectively [94]. Inspired by the work of Mo-based electrodes in lithium-ion batteries, Xu et al. adopted electrochemical activation method to prepare the MoO_2_/Mo_2_N composite nanobelts [95]. They discovered that the MoO_2_ grains gradually in situ formed in the Mo_2_N matrix during the continually electrochemical activation cycling. The combination of MoO_2_ and Mo_2_N can overcome the intrinsic low conductivity and structural degradation of Mo-based materials (Fig. 7a). Specifically, the Mo_2_N matrix can protect the inner MoO_2_ grains from structural degradation during the intercalation of zinc ions. And the small MoO_2_ grains can accommodate more intercalated zinc ions. The synergic effects of MoO_2_ and Mo_2_N lead to significantly improved electrochemical performance. At the high rate of 1 A g^−1^, the formed MoO_2_/Mo_2_N composite nanobelts can still deliver a high discharge capacity of 113 mAh g^−1^ for over 1000 cycles, exhibiting excellent performance at high rate (Fig. 7b, c). Moreover, the electrochemical activation phenomenon was also observed in MnO-based ZIBs. As reported by Kang et al., the charging process leads to gradual formation of porous MnO_2_ nanosheets surrounding MnO particles [96]. The activated layered MnO_2_ nanosheets show significantly improved electrochemical performance. At a specific current of 0.1 A g^−1^, the specific capacity of the electrode can be enhanced to 330 mAh g^−1^.

Xu et al. further studied MoS_2_ with defects induced for application as cathode material in ZIBs with both experimental and theoretical methods [97]. They found that MoS_2−*x*_ nanosheets with tremendous sulfur vacancies can accommodate preferential intercalation of Zn^2+^ ions, showing much higher capacity than the MoS_2_ without defects. Material characterization and theoretical modeling results revealed that interlayer sulfur vacancies and edge sites of MoS_2_ nanosheets can accommodate the intercalated Zn^2+^ ions, leading to a significantly enhanced capacity than MoS_2_ without defects. At the current density of 1 A g^−1^, the defect-engineered MoS_2−*x*_ can deliver a high capacity of 88.6 mAh g^−1^ for over 1000 cycles (capacity retention of 87.8%). Subsequently, Zhi et al. prepared MoS_2_ with expanded interlayer spacing as promising cathode for flexible ZIBs [98]. They investigated the Zn^2+^ intercalation/deintercalation process via XRD and Raman techniques. At the current density of 0.1 A g^−1^, the E-MoS_2_ electrode can deliver a high specific capacity of 202.6 mAh g^−1^ for 600 cycles. Moreover, they further developed a quasi-solid Zn/E-MoS_2_ battery by using polyacrylamide (PAM) polymer as electrolyte. The quasi-solid Zn/E-MoS_2_ battery exhibits good performance under kinds of deformations, demonstrating potential to be applied in next-generation flexible devices. Recently, aqueous zinc–iodine batteries are emerging as an attractive electrochemical energy storage technology due to their low cost, abundant resource, and high energy density [99–102]. Similar to the widely studied lithium–sulfur batteries, zinc–iodine batteries have the similar problems of low reversibility and limited cycling, which is caused by the poor conductivity and soluble active species in the electrolytes. To address these issues, many efforts have been devoted to design iodine/carbon composite materials. For example, Pan et al. prepared microporous carbon with iodine encapsulated inside via solid–liquid conversion reactions [99]. The resulted zinc–iodine/C battery can provide a high capacity of 174.4 mAh g^−1^ at 1 C for over 3000 cycles. Yuan et al. designed the composite material with I_2_ confined in porous carbon cloth. With fully utilizing the active I_2_ material in the composite architecture, the zinc–iodine batteries yield a high energy density of ~ 151 Wh kg^−1^ with long cycling life for 1500 cycles [100].

### Zinc-Ion Batteries based on Nonaqueous Electrolytes

Initially, there was research focused on using room temperature ionic liquid electrolytes (trifluoromethanesulfonic imide, trifluoromethanesulfonic imide, etc.) as electrolytes in zinc-ion batteries owing to their very low vapor pressure, extremely high chemical/physical stability, and high ionic transportation. However, the discharge capacities and cycling lifetime of batteries based on these electrolytes are not satisfactory. To achieve an ideal electrolyte with good electrode compatibility, much work concerning acetonitrile electrolytes has been carried out [103–106]. For example, Zn(ClO_4_)_2_ acetonitrile electrolyte was employed together with NiHCF as cathode in ZIBs [103]. At a rate of 0.2 °C, the battery can deliver a discharge capacity of 55.6 mAh g^−1^ with an average potential of 1.19 V. Gewirth et al. then explored a new series of spinels, ZnNi_*x*_Mn_*x*_Co_2−2*x*_O_4_, as cathode electrodes for ZIBs with organic electrolyte [104]. The full cells were fabricated with these spinels paired with zinc metal anode. At a specific current of 42 mA g^−1^, the batteries can supply a specific capacity of 174 mAh g^−1^ for 200 cycles, corresponding to an energy density as high as 305 Wh kg^−1^. The XRD and EDS results verified the reversible intercalation/deintercalation of Zn^2+^ ions in spinel materials. Meanwhile, the ex situ XPS results confirmed that the reversible conversion between Mn^4+^/Mn^3+^, Ni^4+^/Ni^3+^/Ni^2+^, Co^4+/^Co^3+^ oxidation states took place in spinel cathodes during the electrochemical process. All the results indicate that co-substitution of Mn and Ni for Co in ZnCo_2_O_4_ is an efficient way to promote the intercalation of zinc ions and improve the capacity. As such, the acetonitrile–Zn(CF_3_SO_3_)_2_ electrolyte shows a high anodic stability and relatively low overpotential, leading to an excellent Coulombic efficiency of the Zn anode, while V-based materials usually show low specific capacity and poor rate performance in organic electrolytes [106]. This is because the insertion of Zn ions needs a desolvation penalty at the electrode/electrolyte interface. In the water-based solution, the co-intercalation of water molecules can effectively facilitate the insertion of Zn ions and the penalty is very low. However, the co-intercalation with Zn^2+^ ions in nonaqueous solutions is difficult to be realized due to large radius of the solvation molecules, which is the main reason that ZIBs consisting of nonaqueous electrolytes often show poor kinetics.

## Summarization of Representative Cathode Materials

**Fig. 8:**
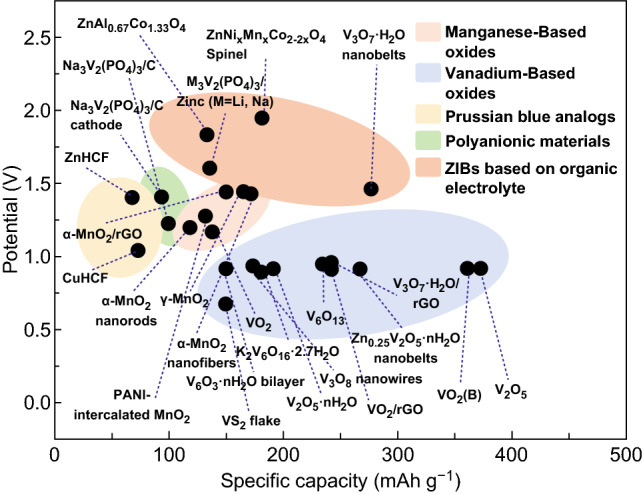
Specific capacity versus discharge potential of various cathode materials for ZIBs

Table 1 summarizes recent developments of some representative cathode materials, including the electrolyte components, testing voltage and current, discharge capacity, and cycling life. And Fig. 8 displays the specific capacity versus the discharge potential for various cathodes. As we can see in Table 1 and Fig. 8, manganese oxides show high operation voltages and acceptable rate capabilities. However, manganese oxides suffer from limited cycling life, due to the dissolution of Mn^2+^ ions during cycling caused by the Jahn–Teller effect during the phase transformation with the intercalation of zinc ions. Mn^2+^ additives in the electrolyte can hinder the dissolution of MnO_2_ electrodes. But appropriate concentration still needs to be examined to balance the Mn^2+^ dissolution and the re-oxidation. Compared with manganese oxides, vanadium oxides exhibit much lower discharge potential, but show enhanced rate performance and prolonged cycling life. The stable layered framework and structural water molecules in the V-based cathodes facilitate fast diffusion of zinc ions, leading to their high rate capability and long cycling life. However, the average operation voltage of vanadium oxides is only about 0.8 V in aqueous ZIBs, which seriously restricts its practical applications. The discharge potential of vanadium oxides can be increased by introducing polyanions or fluorines. For example, M_3_V_2_(PO_4_)_3_ (M = Li, Na) and Na_3_V_2_(PO_4_)_2_F_3_ show much higher discharge potential of about 1.5 V. However, these additional groups lead to increased molecular mass and decreased specific capacities (only 113.5 mAh g^−1^ for M_3_V_2_(PO_4_)_3_ (M = Li, Na), and 50 mAh g^−1^ for Na_3_V_2_(PO_4_)_2_F_3_). Therefore, further explorations should be focused on enhancing both the operating voltage and specific capacity for vanadium-based cathodes. Compared with manganese oxides and vanadium-based cathodes, the electrochemical performance of PBAs is poor. Even though the PBAs can support a high average operation voltage up to 1.5 V [89], they suffer low specific capacities (50–80 mAh g^−1^) and limited cycling life (~ 200 cycles). The poor performance of PBAs is attributed to the randomly distributed Fe(CN)_6_ vacancies that can break the electronic conduction between Fe–CN–M bonds and thus result in poor rate capability. Therefore, it is important to reduce the lattice defects to improve the performance. Moreover, herein we also summarize the ZIBs with aqueous and nonaqueous electrolytes. The anions and solvents in the electrolytes are significant for the diffusion of charge carriers and stabilizing electrode materials. ZnSO_4_ and Zn(CF_3_SO_3_)_2_ solutions are commonly used as electrolytes in aqueous ZIBs due to their excellent electrochemical performances. However, the acidic condition may damage the long-term stability of the Zn metal anode. Compared to ZIBs with aqueous electrolytes, ZIBs with organic electrolytes exhibit higher operation voltages and moderate discharge capacities, but they show much poorer rate performance and limited cycling life, which probably attributes to faster ionic diffusion rate, and higher reversibility of metal deposition/dissolution in mild aqueous electrolytes.

**Table 1:** Summary of electrochemical properties of cathode materials for ZIBs

Cathode	Electrolyte	Operating voltage (V)	Current rate	Capacity (mAh g^−1^)	Cycle performance	Refs.
α-MnO_2_ nanorods	Aqueous Zn(CF_3_SO_3_)_2_	0.8–1.8	5 A/g	115.9	97.7% retention after 4000 cycles	[35]
α-MnO_2_/rGO	Aqueous ZnSO_4_	1.0–1.9	3 A/g	145.3	94% retention after 3000 cycles	[40]
γ-MnO_2_	Aqueous ZnSO_4_	1.0–1.8	0.5 mA/cm^2^	158 mAh/cm^2^	37% retention after 40 cycles	[58]
α-MnO_2_ nanofibers	Aqueous ZnSO_4_	1.0–1.85	1520 mA/g	160	92% retention after 5000 cycles	[60]
MnO_2_	Aqueous ZnSO_4_	1.0–1.8	1885 mA/g	50–70	10,000 cycles	[61]
PANI-intercalated MnO_2_	Aqueous ZnSO_4_	1.0–1.8	2 A/g	125	5000 cycles	[63]
V_2_O_5_	Aqueous Zn(CF_3_SO_3_)_2_	0.2–1.6	5 A/g	372	91.1% retention after 4000 cycles	[65]
Bilayer V_2_O_5_·*n*H_2_O	aqueous Zn(CF_3_SO_3_)_2_	0.2–1.6	6 A/g	~ 200	71% retention after 900 cycles	[66]
Zn_0.25_V_2_O_5_·nH_2_O nanobelts	Aqueous ZnSO_4_	0.5–1.4	2400 mA/g	260	80% retention after 1000 cycles	[67]
VO_2_ (B)	Aqueous Zn(CF_3_SO_3_)_2_	0.3–1.5	100 mA/g	357	50 cycles	[68]
VO_2_	Aqueous Zn(CF_3_SO_3_)_2_	0.7–1.7	10 A/g	133	79% retention after 10,000 cycles	[69]
V_6_O_13_·*n*H_2_O	Aqueous Zn(CF_3_SO_3_)_2_	0.2–1.4	5 A/g	~ 150	1000 cycles	[70]
V_6_O_13_	Aqueous Zn(CF_3_SO_3_)_2_	0.2–1.5	4 A/g	~ 240	92% retention after 2000 cycles	[71]
V_3_O_7_·H_2_O/rGO	Aqueous ZnSO_4_	0.3–1.5	1500 mA/g	245	79% retention after 1000 cycles	[72]
VO_2_/rGO	Aqueous Zn(CF_3_SO_3_)_2_	0.3–1.3	4 A/g	240	99% retention after 1000 cycles	[42]
VS_2_ flake	Aqueous ZnSO_4_	0.4–1.0	200 mA/g	125	99.7% retention after 250 cycles	[47]
H_2_V_3_O_8_ nanowires	Aqueous Zn(CF_3_SO_3_)_2_	0.2–1.6	5 A/g	173.6	94.3% retention after 1000 cycles	[73]
H_2_V_3_O_8_ nanowires/GO	Aqueous Zn(CF_3_SO_3_)_2_	0.2–1.6	6 A/g	270	87% retention after 2000 cycles	[74]
Li^+^ intercalated V_2_O_5_·*n*H_2_O	Aqueous ZnSO_4_	0.4–1.4	10 A/g	192	1000 cycles	[75]
LiV_3_O_8_	Aqueous ZnSO_4_	0.6–1.2	133 mA/g	~140	65 cycles	[76]
K_2_V_6_O_16_·2.7H_2_O nanorod	Aqueous Zn(CF_3_SO_3_)_2_	0.4–1.4	6 A/g	188	82% retention after 500 cycles	[77]
Na_0.33_V_2_O_5_	Aqueous Zn(CF_3_SO_3_)_2_	0.2–1.6	1.0 A/g	218.4	93% retention after 1000 cycles	[78]
NaV_3_O_8_	Aqueous ZnSO_4_	0.3–1.25	4 A/g	165	82% retention after 1000 cycles	[79]
Na_2_V_6_O_16_·1.63H_2_O	Aqueous Zn(CF_3_SO_3_)_2_	0.2–1.6	5 A/g	158	90% retention after 6000 cycles	[80]
Na_1.1_V_3_O_7.9_@rGO	Aqueous Zn(CF_3_SO_3_)_2_	0.4–1.4	300 mA/g	171	100 cycles	[81]
Ca_0.25_V_2_O_5_·*n*H_2_O	Aqueous ZnSO_4_	0.6–1.6	~ 20 A/g	~ 70	96% retention after 3000 cycles	[83]
Ag_0.4_V_2_O_5_	Aqueous ZnSO_4_	0.4–1.4	20 A/g	144	4000 cycles	[84]
NH_4_V_4_O_10_	Aqueous ZnSO_4_	0.4–1.4	10 A/g	255.5	1000 cycles	[85]
ZnHCF	Aqueous ZnSO_4_	0.8–1.9	300 mA/g	68	85% retention after 200 cycles	[41]
CuHCF	Aqueous ZnSO_4_	0.45–1.4	60 mA/g	~50	100 cycles	[86]
CuHCF	Aqueous ZnSO_4_	0.2–1.1	10 C	~40	~80% retention after 1000 cycles	[88]
ZnHCF	Aqueous ZnSO_4_	0.8–1.9	300 mA/g	52.5	81% retention after 100 cycles	[89]
LiV_2_(PO_4_)_3_	Aqueous Zn(OTf)_2_	0.2–1.9	1500 mA/g	~110	78.8% retention after 4000 cycles	[90]
Na_3_V_2_(PO_4_)_3_/C cathode	CH_3_COOLi + Zn(CH_3_COO)_2_	0.8–1.7	50 mA/g	84	68% retention after 200 cycles	[91]
M_3_V_2_(PO_4_)_3_/Zinc (M = Li, Na)	Li_2_SO_4_–ZnSO_4_ aqueous electrolyte	0.7–2.1	0.2 °C	113.5	84.1% retention after 200 cycles	[92]
Na_3_V_2_(PO_4_)_2_F_3_	Aqueous Zn(CF_3_SO_3_)_2_	0.8–1.9	1 A/g	50	4000 cycles	[93]
Na_3_V_2_(PO_4_)_3_/C	Aqueous Zn(CF_3_SO_3_)_2_	0.8–1.7	50 mA/g	97	74% retention after 100 cycles	[43]
MoO_2_/Mo_2_N nanobelts	Aqueous Zn(CF_3_SO_3_)_2_	0.25–1.35	1 A/g	113	78.8% retention after 1000 cycles	[95]
MoS_2_	Aqueous Zn(CF_3_SO_3_)_2_	0.25–1.25	1 A/g	88.6	87.8% retention after 1000 cycles	[97]
MoS_2_	Aqueous ZnSO_4_	0.3–1.5	1 A/g	161.7	97.7% retention after 500 cycles	[98]
Quinones	Aqueous Zn(CF_3_SO_3_)_2_	0.2–1.8	500 mA/g	About 120	87% retention after 1000 cycles	[44]
Polyaniline	Aqueous Zn(CF_3_SO_3_)_2_	0.5–1.5	5 A/g	82	92% retention after 3000 cycles	[52]
NiHCF	Zn(ClO_4_)_2_ acetonitrile electrolyte	0.7–1.8	11.2 mA/g	~56	20 cycles	[103]
ZnNi_x_Mn_x_Co_2–2x_O_4_ Spinel	Zn(OTf)_2_ in MeCN	0.8–2.15	42 mA/g	180	81.66% retention after 200 cycles	[104]
ZnAl_0.67_Co_1.33_O_4_	Zn(OTf)_2_ in MeCN	1.4–2.2	32 mA/g	134	85.1% retention after 100 cycles	[105]
V_3_O_7_·H_2_O nanobelts	ZnOTf) in acetonitrile	0.5–1.8	3000 mA/g	~ 270	80% retention after 200 cycles	[106]

## Solid-state Zinc-Ion Batteries

The ever-increasing demands for portable and wearable electronics have stimulated research interests in flexible rechargeable batteries with excellent electrochemical performances and low cost. As a promising new energy storage system, solid-state zinc-ion batteries (SZIBs) exhibit a series of noticeable advantages, such as high safety without electrolyte leakage, good flexibility, and low cost. Though much work has been done on zinc-ion batteries with liquid electrolytes, progress on SZIBs is still limited due to the lack of high zinc-ion conducting solid-state electrolytes. Therefore, it is imperative to explore the physicochemical properties of suitable solid-state electrolytes and the zinc-ion migration mechanism inside the solid-state electrolytes, which may provide more insight into the realizations of practical SZIBs [30].

**Fig. 9:**
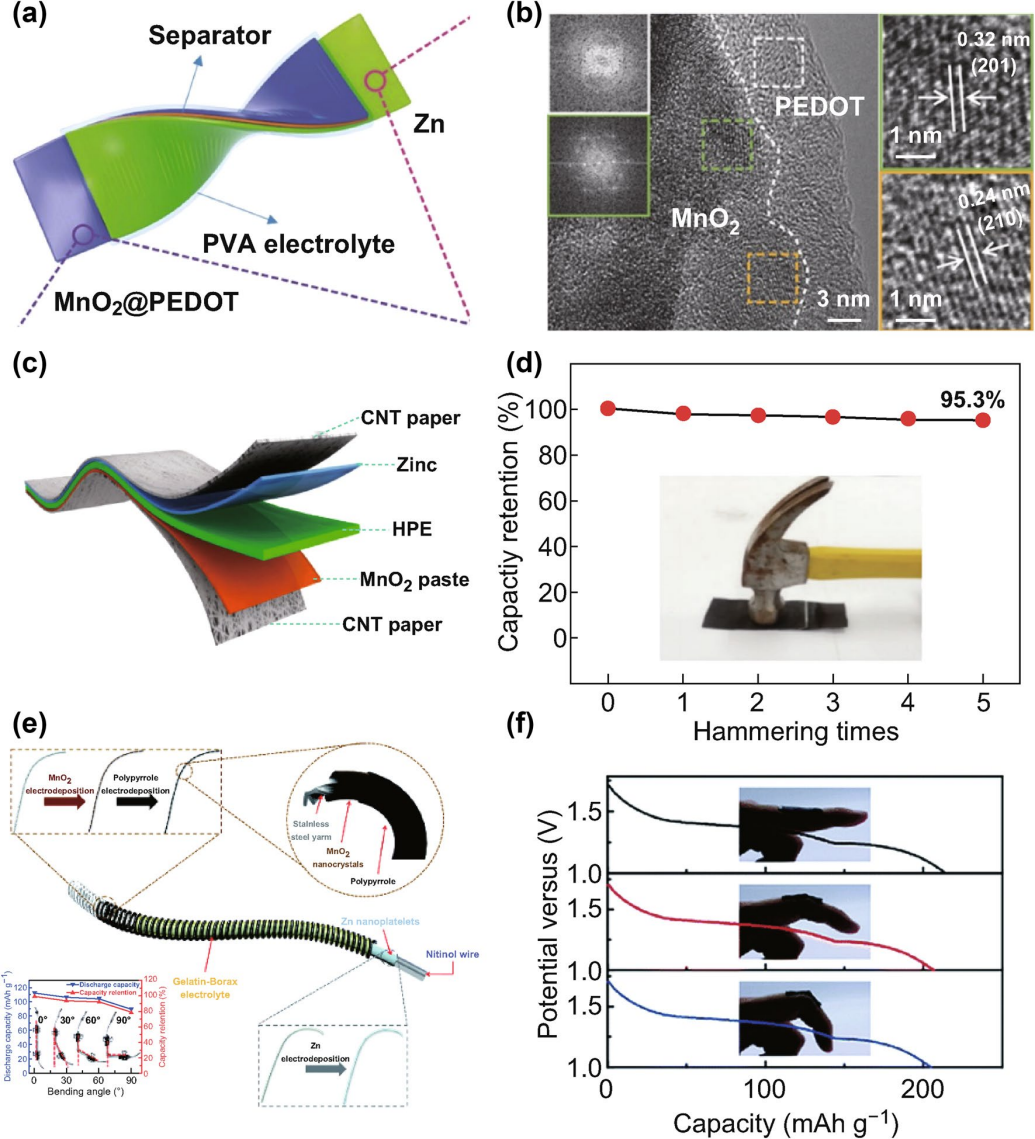
**a** Schematic showing the flexible quasi-solid-state Zn-MnO_2_@PEDOT batteries. **b** HRTEM images of MnO_2_@PEDOT sample [107]. **c** Schematic illustration of the structure of the solid-state ZIB and **d** the hammering test [108]. **e** Schematic illustration of the structure of the wire battery, inset showing the charging–discharging curves corresponding to the wire batteries bending at different angles [109]. **f** Galvanostatic discharge curves of Zn–MnO_2_ batteries bending at different curvatures [110]. With permission from The Royal Society of Chemistry

In 2017, Tong et al. first developed flexible quasi-solid-state Zn–MnO_2_/PEDOT battery with high performance via using PVA/ZnCl_2_/MnSO_4_ gel electrolyte and MnO_2_/PEDOT cathode (Fig. 9a, b) [107]. The as-fabricated battery exhibits a high capacity of 282.4 mAh g^−1^ at 0.37 A g^−1^, durable cycling up to 300 cycles with capacity retention of 77.7%, and a decent energy density of 504.9 Wh kg^−1^ with a power density of 8.6 kW kg^−1^. Moreover, this flexible battery can maintain similar discharge properties and show no capacity loss under bending or twisting, demonstrating excellent mechanical strength. However, it should be noted that the quasi-solid-state battery usually presents relatively lower rate capability compared to the aqueous system, attributed to the low ionic conductivity and high charge transfer resistance of the PVA/ZnCl_2_/MnSO_4_ gel electrolyte.

Zhi et al. also developed a wearable solid-state zinc-ion battery with high safety via utilizing novel gelatin–PAM-based solid-state polymer electrolyte with ZnSO_4_ and MnSO_4_, *α*-MnO_2_@CNT cathode, and flexible Zn foil anode (Fig. 9c) [108]. The solid-state polymer electrolyte was prepared by filling gelatin–PAM chains in the PAN fiber network. Such highly porous interconnected framework of the solid-state polymer electrolyte displays an extremely high ionic conductivity of 1.762 × 10^−2^ S cm^−1^ and desirable mechanical strength of 7.76 MPa. Benefiting from the solid-state electrolyte with high ionic conductivity, the zinc-ion battery cell exhibits an impressive reversible capacity of 306 mAh g^−1^ at a specific current of 61.6 mA g^−1^ and decent capacity retention of 97% up to 1000 cycles at 2772 mA g^−1^. Even under various severe conditions such as bending, hammering (Fig. 9d), cutting, washing, combustion, weight loading, sewing, and drilling, the designed solid-state zinc-ion batteries show desirable electrochemical behavior and high stability, demonstrating great potential as new generation energy storage system to power flexible and wearable electronics.

Zhi et al. further designed a smart wire-shaped flexible zinc-ion battery with shape memory function. The designed batteries show improved electrochemical performances with unique shape memory function by utilizing the temperature-initiated shape memory effect (Fig. 9e) [109]. The gel polymer electrolyte is fabricated by employing gelatin and borax in aqueous solution of ZnSO_4_/MnSO_4_, and it presents higher ionic conductivity of 2.0 × 10^−2^ S cm^−1^ compared to the bare gelatin gel electrolyte with 1.39 × 10^−2^ S cm^−1^. Owing to the introduction of borax as a cross-linker into the gelatin gel electrolyte, the ionic conductivity, water retention capability, and mechanical strength of the gelatin–borax gel electrolyte are effectively improved, thereby enhancing the electrochemical performances of the zinc-ion battery. Such flexible wire battery exhibits a specific capacity of 174.2 mAh g^−1^ at 0.5 °C and durable cycling performance up to 1000 cycles with corresponding Coulombic efficiencies over 99%. Additionally, its electrochemical performance can be well preserved when bent to 90° or consecutively bent to 45° for over 500 cycles, indicating its superior resistance against mechanical deformation.

Searching for a stable sulfate-tolerant polymer electrolyte without polymer precipitation, Li et al. explored a xanthan biopolymer in combination with aqueous ZnSO_4_/MnSO_4_ solution to fabricate a very stable gum bio-electrolyte, which was then assembled in a rechargeable Zn/MnO_2_ battery for electrochemical characterizations [110]. Owing to its favorable molecular structure composed of α,β-1,4-linked glucan backbone with trisaccharide side chains connected with backbone residues by α-1,3 linkages, the xanthan gum shows high salt tolerance stability. The gum bio-electrolyte is highly conductive (1.46 × 10^−2^ S cm^−1^ at room temperature and 2.5 × 10^−3^ S cm^−1^ at − 8 °C), and its ionic conductivity remains unchanged even after 1-year storage, indicating its ability of working in a wide temperature range for the long term. As a result, this battery presented a specific capacity of 260 mAh g^−1^ at 1 °C, high rate capability, good cycling performance with 90% capacity retention after 330 cycles at 1 °C, and even maintained a decent capacity of 127 mAh g^−1^ up to 1000 cycles at 5 °C. Moreover, the battery cell exhibits outstanding durability under bending and twisting (Fig. 9f) and effectively prohibits the zinc dendrite growth during continuous charge–discharge cycles, suggesting that the gum bio-electrolyte is a very promising candidate for flexible energy storage systems.

**Fig. 10:**
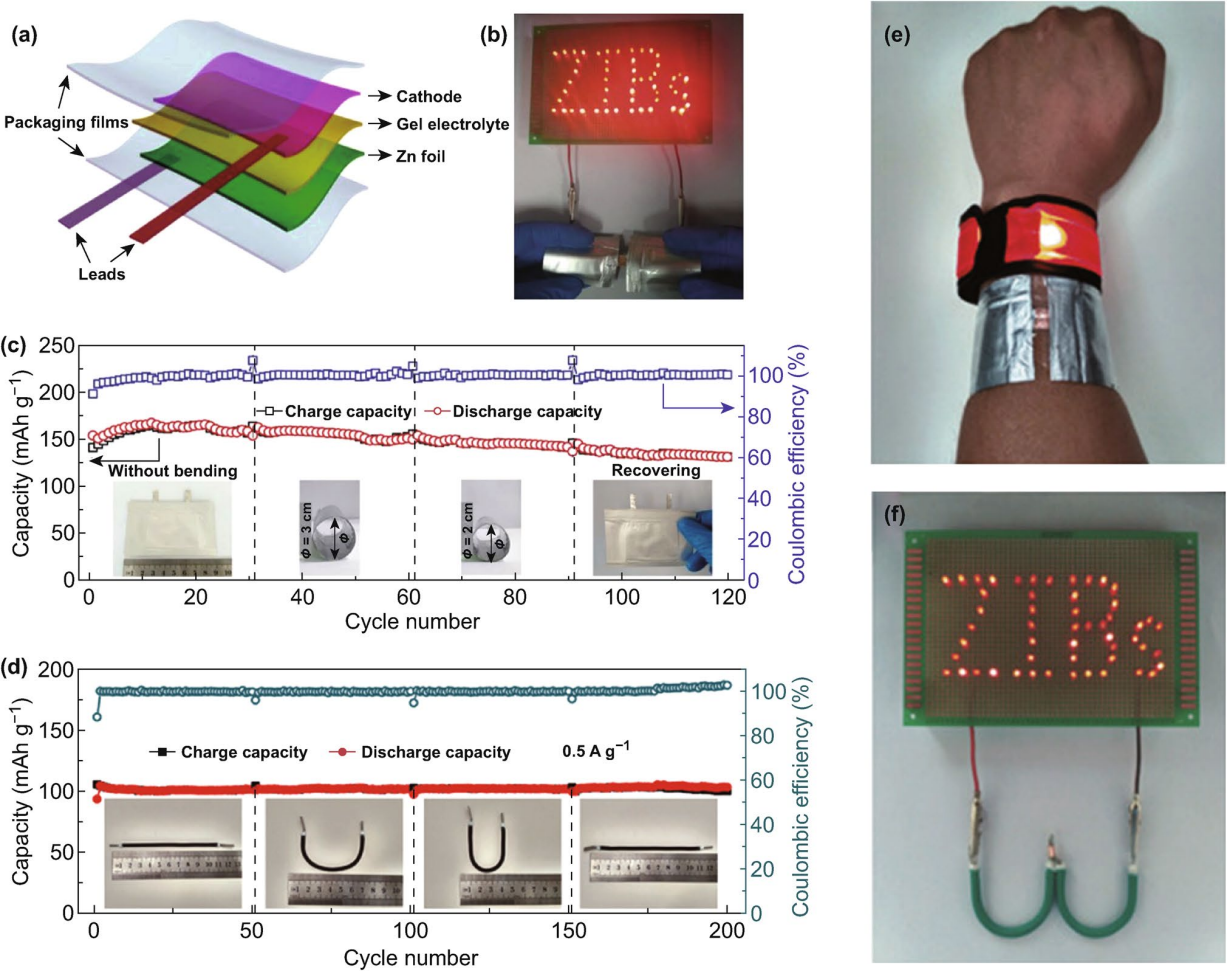
**a** Schematic diagram of a quasi-solid-state Zn/NVO battery [50], **b** LED array with 52 bulbs powered by two quasi-solid-state Zn/NVO battery under bending condition, **c** cycling performance under various bending states at 0.5 A g^−1^ of the flexible quasi-solid-state Zn/NVO battery. **d** cycling performance of soft-packaged and cable-type quasi-solid-state batteries under various bending states at 0.5 A g^−1^. **e** A wrist strap powered by two soft-packaged quasi-solid-state batteries in series, **f** an LED array powered by two cable-type quasi-solid-state batteries in series [52]. With permission from Elsevier and The Royal Society of Chemistry

Inspired by Zhi et al.’s work above, Chen et al. investigated a Zn/NaV_3_O_8_·1.5H_2_O (NVO) cell by using a quasi-solid-state electrolyte that consists of gelatin and aqueous ZnSO_4_ solution (Fig. 10a) [50]. Although the preparation method is simple, the gel electrolyte shows degraded ionic conductivity compared to the corresponding aqueous part. Even so, the flexible quasi-solid-state Zn/NVO battery still delivers a good rate capability of 288, 228, 160, 115, and 80 mAh g^−1^ at 0.1, 0.2, 0.5, 1, and 2 A g^−1^, respectively. Furthermore, such flexible energy storage system can maintain the capacities under various bending states during charge–discharge process with only a slight capacity loss, indicating high stability of the quasi-solid-state cell (Fig. 10c). In addition, two quasi-solid-state Zn/NVO cells under bending condition in series can light up the LED array of 52 bulbs, suggesting that they can be great candidates for practical flexible energy storage devices, as shown in Fig. 10b.

Chen et al. also developed a flexible quasi-solid-state batteries by using polyaniline (PANI) as cathode, PVA/Zn(CF_3_SO_3_)_2_ gel as electrolyte, and Zn metal as anode (Fig. 10d–f) [52]. Such soft-packaged quasi-solid-state Zn/PANI battery delivers a reversible capacity 109 mAh g^−1^ in the first cycle at 0.5 A g^−1^ and 91.7% capacity can be retained after 200 cycles, while the cable-type battery displays a capacity of 106 mAh g^−1^ for the first discharge process at 0.5 A g^−1^ and 91.5% capacity retention can be achieved over 200 cycles (Fig. 10d). The highly stable electrochemical performances of both soft-packaged and cable-type Zn/PANI batteries under different bending states hold great promise for flexible electronic applications. Moreover, two soft-packaged and two cable-type flexible cells can successfully light up a wristwatch and an LED array under bending condition, respectively (Fig. 10e, f).

There has been another report concerning quasi-solid-state zinc-ion battery with high rate capability using a layered zinc orthovanadate array as cathode, a Zn array as anode, and a gel electrolyte composed of fumed silica with aqueous ZnSO_4_ solution [111]. This battery displays superior electrochemical behaviors and ultra-stable flexibility. Specifically, the flexible cell exhibits a highly reversible capacity of 204 mAh g^−1^ with an initial high Coulombic efficiency of 95% at 0.5 °C, corresponding to the two-electron transfer process. Due to the nanoarray structure in both electrodes providing short fast ion migration pathways and introduced insertion pesudocapacitance, the quasi-solid-state battery delivers an excellent rate capability of 160 mAh g^−1^ at 10 °C and 101 mAh g^−1^ even at 50 °C. Furthermore, the quasi-solid-state ZIB shows long-term cycling stability for 2000 cycles at 20 °C and considerable energy density of 115 Wh kg^−1^ with a power density of 5.1 kW kg^−1^ based on the total masses of cathode, anode, and current collectors. Additionally, the battery reveals no obvious capacity fading with over 96% capacity retention under continuous 100 bending cycles, showing its high tolerance of bending without deterioration of the discharge performance.

**Fig. 11:**
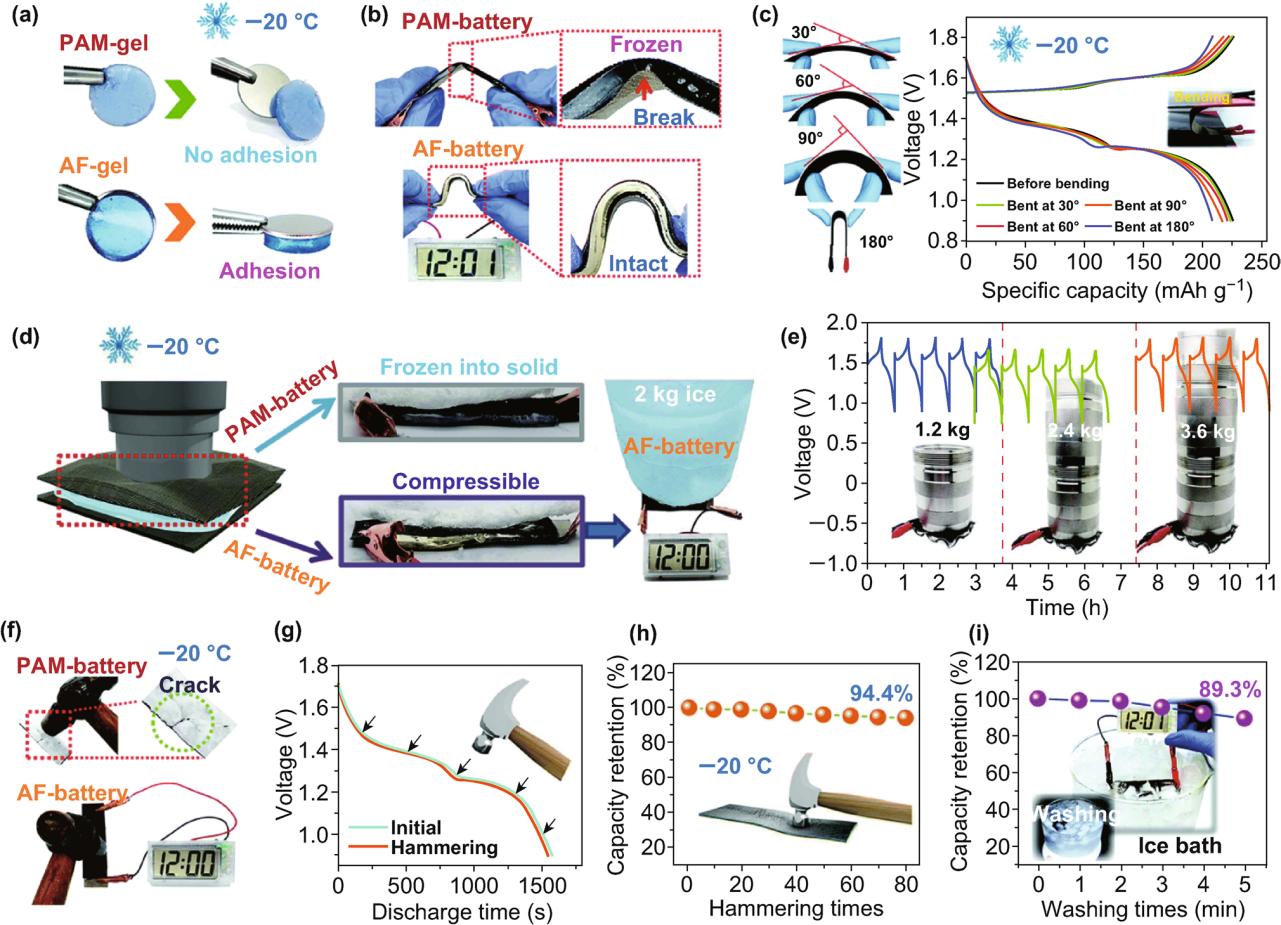
a Comparisons of the adhesion force of the PAM gel and AF gel at − 20 °C. **b** Bending test of the PAM battery and AF battery at − 20 °C. **c** Charge–discharge profile of the AF battery under bending test at 0.2 A g^−1^. **d** PAM battery and AF battery holding an ice block; the AF battery can still power an electrical watch at − 20 °C. **e** Cycling test of the AF battery under various heavy loads at − 20 °C. **f** Hammering tests of the PAM battery and AF battery after 1 day of cooling at − 20 °C. **g** Discharge curves and **h** cycling test of the AF battery under hammering tests. **i** Capacity retention of the AF battery washing in an ice bath [113]. With permission from The Royal Society of Chemistry

Recently, Zhi et al. obtained a flexible self-healing solid-state aqueous rechargeable NiCo/Zn battery by employing NiCo hydroxide as cathode, flexible Zn/carbon cloth as anode, and Fe^3+^-cross-linking sodium polyacrylate (PANa-Fe^3+^) hydrogel as solid-state electrolyte [112]. This self-healing electrolyte displays enhanced healing property and good ionic conductivity owing to the Fe^3+^ cross-linkers forming ionic bonds between the PANa chains, as well as the favorable compatibility of PANa-Fe^3+^ with aqueous solution of Zn(CH_3_COO)_2_ + KOH. Hence, the assembled NiCo/Zn batteries with such intrinsically self-healing PANa-Fe^3+^ electrolytes show autonomically self-healing ability with over 87% capacity retention after four cycles of cutting/healing. When the two broken parts of the cell were brought to connect, the watch was on again without weakening the brightness, indicating excellent healing performance and high potential applications in broken electronics. Moreover, a flexible Zn/MnO_2_ battery with exceptionally electrochemical performances has been achieved and can operate at subzero temperatures (Fig. 11) [113]. Hydrogel electrolytes are attractive for flexible ZIBs by virtue of their safety and high physical/chemical stability. However, the freezing issue of hydrogel electrolytes under subzero temperatures would lead to severe capacity fading and elasticity degradation, in addition to loss of mechanical robustness and flexibility under cold condition. In this work, a strong hydrogen bonding with water anchored the water units within the polymer electrolyte was designed, rendering superior anti-freezing property even at − 20 °C. The constructed Zn/MnO_2_ flexible battery maintained high electrochemical performances, impressive durability, and flexibility even being compressed, bent, or washed in an ice bath at − 20 °C (Fig. 11e–i). Such an excellent polymer electrolyte allows flexible ZIBs to be used under extreme conditions, such as aerospace or submarines.

## Design of Zinc Anodes and Separators

Most studies in ZIBs employ commercial zinc foil as the anode for investigation of ZIBs. Although zinc metal is considered as a promising anode for aqueous ZIBs owing to its intrinsic safety, low toxicity, and high theoretical capacity, metallic zinc inevitably suffers from passivation, irreversibility, corrosion, and growth of dendrite during plating/stripping. Especially in alkaline aqueous electrolytes, passivation problems caused by the formation of ZnO layer can limit the surface contact between electrolyte and zinc anode and seriously reduce the conductivity of the anode [114, 115]. Moreover, the growth of zinc dendrites can continuously consume water during cycling and irreversibly produce by-products (Zn(OH)_4_^2−^, etc.), leading to very low Coulombic efficiency, poor capacity, limited cycling life of ZIBs. Zinc dendrite still tends to form especially at high current densities. It is believed that the formation of zinc dendrite is due to the uneven Zn^2+^ distribution on flat metallic Zn foil. The sharp end of dendrite tips can act as a charge center in the electric field, supporting further growth of dendrites [54]. Moreover, with the growth of zinc dendrite, the surface area of zinc anode increases, while the corrosion and other surface-dependent reactions increase with the larger surface area of zinc anode, leading to continuously consuming of zinc anode and a faster fading of the battery performance. To overcome these problems, many efforts have been devoted to designing the composite nanostructure of the Zn metal anode, adding additives in electrolyte, or changing the concentration of zinc salts in electrolyte.

Composite nanostructure design is deemed as an effective strategy to restrict dendrite growth, suppress the formation of by-products, and constrain the corrosion of zinc anode [116]. For example, Yan et al. designed lasagna-like nanostructured Zn anode, in which ZnO nanoparticles are wrapped with graphene oxide nanosheets [117]. The composite anode can provide much higher volumetric capacity and capacity retention when compared with pure ZnO. Moreover, Wang et al. deposited zinc on conductive graphite felt via electrodeposition method [118]. The graphite felt substrate can facilitate fast electron transport and efficient zinc plating in various specific directions, leading to enhanced cycling stability.

In addition to the above composite nanostructure design of Zn anodes, adding additives in electrolyte is also an important strategy to constrain the corrosion and dendrite formation of Zn metal anode. For instance, as reported by Mantia et al., the electrolyte’s additive can decrease the grain growth rate and thus affect the morphology of zinc electrodeposition and suppress the formation of dendrites [119]. Unfortunately, at the same time, it can also decrease the kinetics of reaction and cycling stability of ZIBs due to induced higher overpotential. Mantia and his co-workers studied the effect of branched polyethyleneimine (BPEI) as an electrolyte’s additive [119]. They found that the introduction of BPEI can adjust the morphology of the deposited Zn and decrease the size of the crystals, leading to the improved efficiency and stability. Moreover, Chen and his co-workers found that the addition of Na_2_SO_4_ in ZnSO_4_ aqueous electrolyte can effectively hinder the growth of Zn dendrites [79]. This is because sodium ions with a lower reduction potential can form a positively charged electrostatic shield on Zn ions, avoiding the growth of Zn dendrites. In addition, Chen’s group systemically studied the additives in gel electrolyte [120]. They first applied fumed silica as additive in gel electrolyte and found that the fumed silica can significantly reduce dendrite formation. However, the adding of fumed silica can also increase the corrosion on Zn anode. Therefore, they further introduce pyrazole in electrolyte and found that the pyrazole can serve as a good additive to inhibit both zinc corrosion and dendrite growth. The optimized composition 5% fumed silica + 0.2% pyrazole finally exhibits improved performance with enhanced open-circuit voltage, rate capability, and cycling stability.

Highly concentrated neutral Zn-ion electrolyte is another effective method to suppress the dendrite growth. To completely eliminate the formation of Zn dendrites, Wang et al. developed a unique aqueous electrolyte at high concentrations composed of 1 M Zn(TFSI)_2_ and 20 M LiTFSI, proved to achieve 100% Coulombic efficiency and no dendrite formation during Zn plating/stripping [121]. In addition, Chen and his co-workers developed Zn/VOPO_4_ batteries using a water-in-salt electrolyte to realize reversible oxygen redox chemistry in a high voltage region [122]. Since Zn metal anodes show high corrosion current and low positive corrosion potential in 1 M Zn(Tr)_2_ electrolyte, they applied highly concentrated 21 M LiTFSI/1 M Zn(Tr)_2_ water-in-salt electrolyte in ZIBs, which can suppress the corrosion of zinc and also constrain the dissolution of VOPO_4_ cathode due to the limited activity of water. The water-in-salt electrolyte facilitates the reversible oxygen redox reaction in Zn/VOPO_4_ batteries, leading to increased discharge potential to 1.56 V and significantly improved power density from 160 to 217 Wh kg^−1^, which is comparable with lithium-ion batteries. This work of water-in-salt electrolyte is scientifically enlightening for future research. However, the electrolyte with high Zn and Li salts is quite expensive and largely hinders its potential for grid-scale production.

**Fig. 12:**
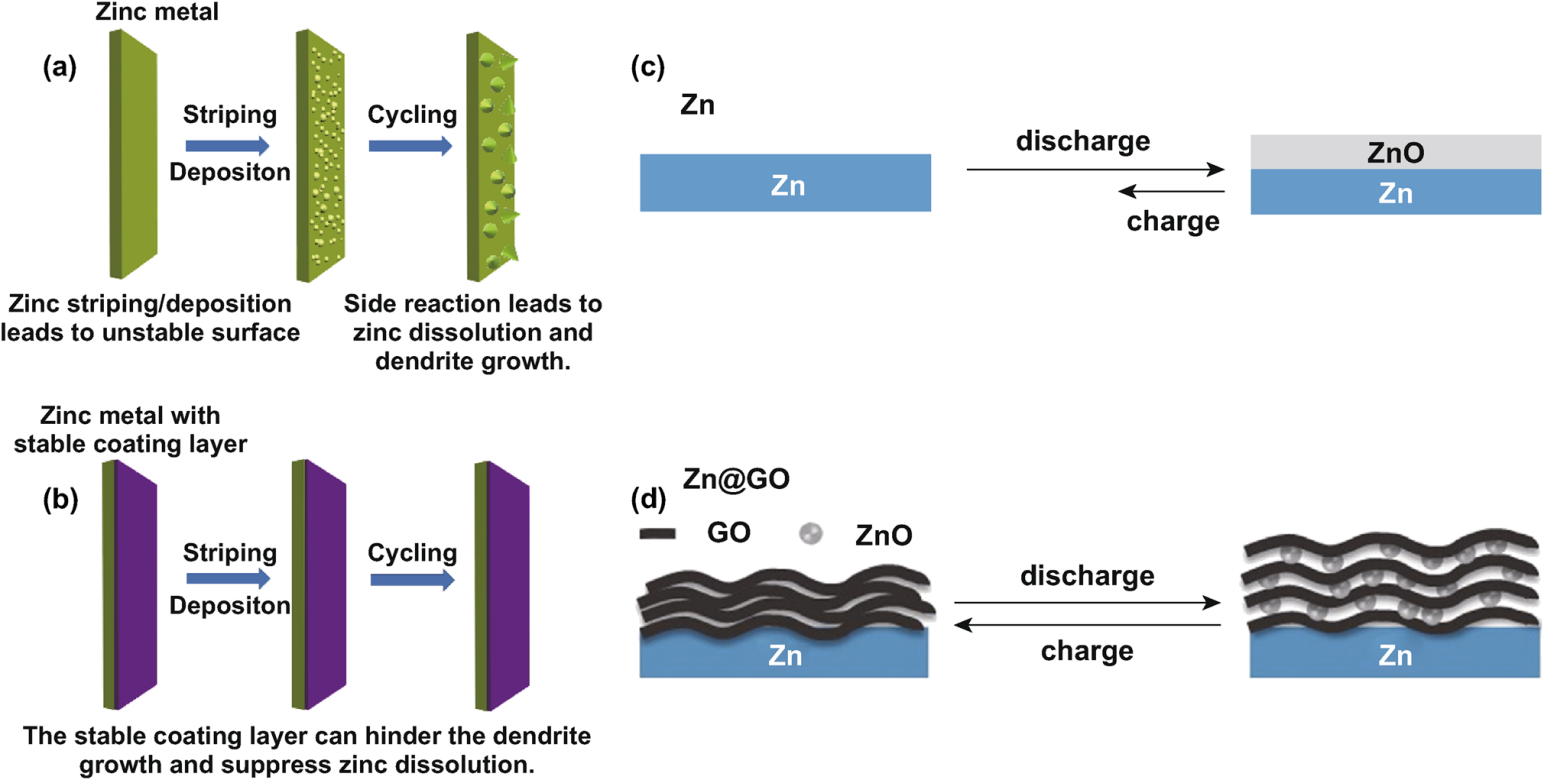
Schematic illustration of the zinc metal anode with stable coating layer. **a** Plating and stripping of zinc ions lead to unstable surface. Side reaction during continuous cycling can lead to passivation and dendrite growth. **b** A thin coating layer leads to a stable deposition/stripping process, hindering dendrite growth, and by-product formation. Schematic of morphological changes about **c** the formation of ZnO passivation layer on Zn anode during charging and discharging. **d** GO on Zn surface can suppress the formation of ZnO passivation and slow down the dissolution of Zn metal [124]. With permission from Elsevier

It is known that the passivation and dendrite growth on zinc anode surface could be reduced in acidic electrolyte [61]. However, acidic electrolytes will cause the corrosion on Zn anode, seriously affecting long-term cyclability. Surface coating on zinc metal foil is an effective way to alleviate the corrosion of zinc anode and thus improve the performance. As shown in Fig. 12a, b, stable coating layer on zinc anode can slow down zinc dissolution and dendrite growth. For example, Zhao et al. applied atomic layer deposition method to coat the zinc metal plate with thin TiO_2_ layer to prevent corrosion during electrochemical reactions [123]. They demonstrated that TiO_2_ layer can effectively protect the inner zinc from corrosion and suppress Zn(OH)_2_ by-product formation, leading to improved capacity retention and prolonged cycling life. In addition, Zhi et al. coated Zn anode with a uniform porous nano-CaCO_3_ layer to enhance the electrostripping/electroplating stability [124]. The porous uniform coatings can guide the Zn plating reaction on the entire Zn anode surface, effectively avoiding the corrosion and the growth of large protrusions/dendrites. Liu et al. coated zinc metal anode with graphene oxide (GO) nanosheets via casting method (Fig. 12c, d) [125]. The GO nanosheets can not only prevent the Zn intermediates from dissolving into aqueous electrolyte, but also improve the surface electron conductivity, leading to the prolonged cycling life and rate capability. Moreover, synthesis of zinc anode with organic additives is another strategy to reduce corrosion. For example, Chen et al. prepared novel zinc anode via electroplating with various organic additives [126]. They found that organic additives can change the surface crystallographic orientation, surface density, and texture of the produced zinc anode. Based on the linear polarization results, compared with the commercial pure zinc metal, the corrosion rate of the prepared Zn-SDS is 30 times lower, showing significantly improved corrosion tolerance. However, there are still very few studies on mechanisms of suppressing corrosion and passivation of zinc anode by now. Therefore, more research efforts should be made to manipulate the Zn^2+^ chemistry based on the Zn anode to overcome the above-mentioned corrosion and dendrite growth problems.

Separator is also critical in ZIBs for practical applications, serving to prevent short circuit by separating the cathode and anode. Currently, most ZIB studies employ filter paper or glass fiber membrane as separators in the battery cells. The systematic explorations on the separator materials are still missing, and it would be helpful to have more research in this regard to realize large-scale utilization of ZIBs. Recently, Liu et al. have constructed a cross-linked polyacrylonitrile-based membrane that is mechanically robust and cost effective, for application as the separator in rechargeable ZIBs [127]. Based on the synthetic merits, Zn/Zn symmetric cells based on this separator exhibit superior long cyclability of over 350 cycles with efficient dendrite growth suppression and lower polarization, which provides an important solution for future design of separators.

## Conclusions

In summary, we provide a timely review on the recent developments in zinc-ion batteries, including the cathode materials, liquid-based electrolytes, and solid-state electrolytes. The preparation methods of the electrode materials, fundamental electrochemical reaction mechanisms, corresponding battery performances, and their correlations are discussed in detail. Continuous efforts are being made for exploring a vast variety of cathode materials. However, there remain many essential electrochemical details that are yet to clarify. For example, the electrochemical reaction mechanisms of Mn-based cathodes in ZIBs are still disputable, with three different mechanisms of Zn^2+^ migration, proton migration, and Zn^2+^/H^+^ co-migration being reported. Moreover, the combination of valence state change of manganese ion during cations insertion and corresponding typical voltage plateaus still need to be examined carefully, which may shed more light on the electrochemical mechanisms and thus provide effective guidance for constructing Mn-based electrode/Zn batteries with improved performances. Compared to the Mn-based electrodes, V-based cathodes display a higher specific capacity and longer cycling performances, but the lower discharge plateaus ~ 0.7 V is a distinct drawback. In addition, V-based materials may continuously dissolve in aqueous acidic Zn^2+^ electrolyte (ZnSO_4_ or Zn(CF_3_SO_3_)_2_ electrolyte). Therefore, developing effective strategies to prevent the vanadium dissolution is necessary, such as adjustment of the electrolyte pH or growth of protective layers on the surface of the V-based cathodes. For the PBA cathodes in ZIBs, removing interstitial water content and reducing crystal defects help to increase the robustness of their crystalline structure and further improve their electrochemical performances.

As to the liquid-based electrolytes, their electrochemical behaviors and compatibility with both cathode and Zn anode are essential for the overall electrochemical properties. Typically, aqueous Zn(CF_3_SO_3_)_2_ and ZnSO_4_ electrolytes are the most commonly used solutions in ZIBs, owing to their better electrochemical performances than other zinc salts. It should be noted that Zn(CF_3_SO_3_)_2_ is much more expensive than ZnSO_4_ (with over 18 times higher price), which is not a cost effective electrolyte choice in large-scale ZIB production, while ZnSO_4_ is accompanied with the formation of unfavorable by-product of hydroxide sulfate. Therefore, it is imperative to investigate the by-product caused by ZnSO_4_ electrolyte and the interface fluctuation between the Zn anode and ZnSO_4_ electrolyte, which may facilitate industrial production of ZIBs based on low-cost ZnSO_4_ electrolytes. For the solid-state electrolyte, most reported polymer electrolytes suffer from low ionic conductivity, insufficient mechanical strength, and fast degradation. Therefore, studies on the chemical conjunction between Zn-based electrolyte and polymer hosts and their joint electrochemical properties will play important roles in advancing solid-state electrolytes with high stability, broad voltage window, and improved long-term cycling performance. Furthermore, the systematic studies of solid-state polymer electrolytes operating in subzero or high temperature are lacking, which hinder the developments of ZIBs for harsh conditions, and more efforts should be made to explore and obtain electrolytes working in a wide temperature range. Despite much progress that has been achieved in the area of ZIBs, there remain challenges as summarized above. Additional fundamental studies are urgently needed, which are crucial for maturing the battery technology and realizing practical zinc-ion batteries for wide applications.

Overall, the combination of environment-friendly property, high safety, high energy density, and long cycling life makes ZIBs high potential candidates for future energy storage devices. Moreover, compared with current LIBs (about $300 per kWh), the price of ZIBs ($65 per kWh) is much lower, which is even comparable with lead acid batteries. The low cost makes ZIBs very promising for practical large-scale applications. The current manufacturing can be leveraged to large-scale commercialization for ZIBs. However, the major challenges of ZIBs still reside in the high energy efficiency cathode, stable Zn anode, and cheap electrolytes. In the near future, adequate zinc anodes, metal oxide cathodes (probably MnO_2_-, V_2_O_5_-based materials), and electrolytes can be designed with more mature technology. The commercialization of rechargeable ZIBs will potentially be scaled up.
